# Projected compositional reorganization of Southern plant assemblages in South Korea under climate scenarios using species distribution models

**DOI:** 10.1038/s41598-026-44558-6

**Published:** 2026-03-14

**Authors:** So-Jin Kim, Chi Hong Lim

**Affiliations:** Department of Bio & Environmental Technology, Seoul Women`s University, 621 Hwarang-ro, Nowon-gu, Seoul, 01797 Korea

**Keywords:** Biodiversity Conservation, Climate Change, Compositional Trajectories, Kernel Density Estimation, Southern Plant Assemblages, Species Distribution Modeling, Ecology, Ecology, Environmental sciences

## Abstract

Understanding how plant communities reorganize under climate change is essential for effective biodiversity conservation and restoration. We developed a spatially explicit, multi-scenario framework to evaluate future dynamics of southern (warm-temperate and subtropical-affiliated) vascular plant assemblages in South Korea. Species distribution models generated projections under three climate scenarios (SSP1–2.6, SSP3–7.0, and SSP5–8.5) across four time periods (1980–2010, 2010–2040, 2040–2070, and 2070–2100). These projections were integrated with spatial environmental clustering, kernel density estimation, and ordination-based trajectory analysis to characterize spatial persistence, compositional change, and directionality. Results revealed strong spatial heterogeneity in community responses. Environmental clusters were classified into stable, transitional, and transformational types based on the magnitude and direction of compositional change and climatic alignment. Multivariate analysis (PERMANOVA) showed that spatial cluster identity explained substantially more variation in community composition than temporal period (R² = 0.364 vs. 0.083). Stable clusters exhibited limited change and strong climatic alignment, whereas transformational clusters showed large shifts and frequent directional misalignment. Transitional clusters displayed intermediate dynamics. By jointly capturing spatial persistence, compositional trajectories, and directional coherence, this framework supports forecasting vegetation responses and climate-resilient conservation planning, with relevance to global biodiversity initiatives such as the Kunming–Montreal Global Biodiversity Framework.

**Supplementary Information:**

The online version contains supplementary material available at 10.1038/s41598-026-44558-6.

## Introduction

Accelerating climate change has intensified concerns over biodiversity loss and ecosystem instability, prompting global efforts to establish forward-looking conservation strategies. The Kunming–Montreal Global Biodiversity Framework (GBF), for example, sets ambitious targets to halt biodiversity loss and restore ecosystem functions by 2030. Its Target 3 calls for the protection of at least 30% of global land and sea areas, while Target 2 emphasizes the restoration of degraded ecosystems and the enhancement of ecological connectivity^1^. However, the implementation of these goals remains grounded in static representations of species distributions and protected area boundaries^2,3^, which insufficiently account for dynamic biogeographic responses to environmental change. Long-term conservation effectiveness will increasingly depend on the ability to identify and protect regions of ecological stability or compositional volatility under future climate scenarios^4^.

The Korean Peninsula is a biogeographic transition zone shaped by complex interactions between mountainous terrain and adjacent ocean currents^5^. The collision of the warm East Korea Warm Current and the cold Yellow Sea Bottom Cold Water creates sharp climatic gradients over short spatial distances^6^, resulting in heterogeneous microclimates that influence plant community dynamics^7^. Southern lineage species (hereafter southern species), in particular, tend to occur in coastal lowlands and warm refugia^8^, and many exhibit high climatic sensitivity with limited capacity for northward or upslope migration^9–11^. Their future responses are thus more likely to involve community-level restructuring, fragmentation, or the formation of novel assemblages, rather than uniform range shifts^12–14^. These dynamics are further shaped by physical barriers and anthropogenic land use, underscoring the need for analysis frameworks that capture compositional change at the community—not just species—level. In this context, the Korean Peninsula provides a particularly informative biogeographic setting under rapid climate change^15–17^.

While species distribution modeling (SDM) has become a dominant tool for predicting climate-driven habitat shifts^18,19^, its utility in detecting community-level reorganization remains limited^11,20,21^. Most SDM-based studies project future distributions for individual species and infer potential overlaps or turnover from aggregated results^18^. However, such approaches rarely quantify the persistence, fragmentation, or directional change in community composition over time. Recent efforts to assess temporal beta diversity have advanced understanding of compositional turnover^19,22^, but often neglect the structural coherence of community types and the extent of their spatial realignment across time and climate scenarios^23–25^. Analytical strategies capable of capturing both the spatial structure and temporal trajectory of assemblages are thus increasingly needed.

In response, this study introduces an integrative framework for modeling climate-induced shifts in plant community structure and distribution. Based on ensemble SDMs for 95 southern plant species at 10 km resolution, we delineate community types using unsupervised clustering of predicted species assemblages. We then trace compositional trajectories across three future climate scenarios, statistically define spatial response zones using richness-weighted presence matrices and evaluate structural reorganization in climate space using a two-dimensional temperature–precipitation niche diagram^26^. This framework enables simultaneous quantification of spatial displacement, internal variation, and climatic realignment of community types. These are key dimensions often neglected in single-species forecasts^25^.

The aim of this study is to characterize the spatial and compositional responses of southern plant communities under changing climates, thereby informing climate-resilient conservation strategies. Specifically, we pursue three objectives: (1) to quantify the degree of temporal reorganization across multiple climate scenarios, capturing how community composition shifts over time; (2) to identify spatial zones of structural persistence or instability, detecting areas likely to remain stable versus those prone to fragmentation or turnover; and (3) to determine whether projected community reassembly is primarily driven by tracking thermal gradients, precipitation constraints, or their combined influence, using climate-space displacement and directional alignment metrics. To address these objectives in an integrated manner, we combine species-level distribution modeling with spatial clustering and multiple complementary analyses that together link projected range changes to emergent, zone-level community dynamics. Rather than prescribing fixed conservation zones, this study proposes a reproducible analytical framework for detecting patterns of community coherence and volatility, serving as a strategic foundation for long-term conservation planning under conditions of ecological uncertainty.

## Materials and methods

### Study site

**Fig. 1:**
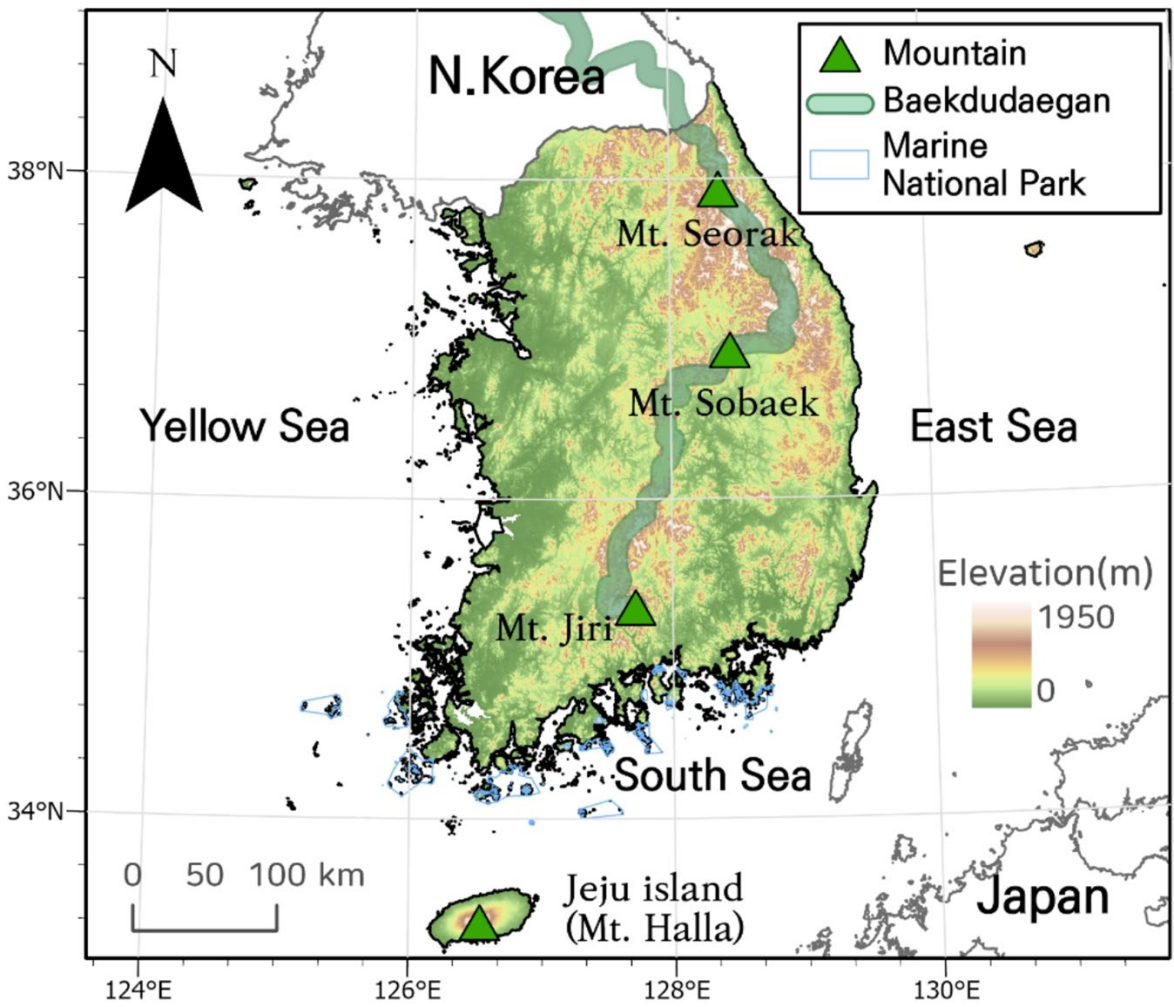
Overview map of the South Korea showing the Baekdudaegan mountain range, major mountains, and islands. The map was generated using ArcGIS Pro version 3.2 (Esri Inc., https://www.esri.com/en-us/arcgis/products/arcgis-pro/overview). Administrative boundaries were obtained from GADM v4.1 (https://gadm.org).

The study area encompasses the entirety of South Korea, including both inland and coastal regions, with elevations ranging from sea level to 1,950 m at Mt. Halla (Fig. 1). To represent the full spatial extent of environmental conditions relevant to southern species distributions, we included a network of inland and coastal protected areas across the peninsula.Coastal protected areas, including Jeju Island, Hallyeohaesang, and Dadohaehaesang National Parks, were included to capture southern coastal environments influenced by maritime conditions^27,28^. In addition, portions of the eastern coastal region were included to account for localized thermal variability associated with regional atmospheric processes^29^. Inland mountain regions, particularly along the Baekdudaegan range, were included to represent elevational gradients relevant to potential altitudinal shifts in species distributions^30^. Collectively, these coastal and inland components provide a continuous spatial framework for assessing geographic patterns and projected changes in southern species distributions across South Korea.

### Analytical framework

**Fig. 2:**
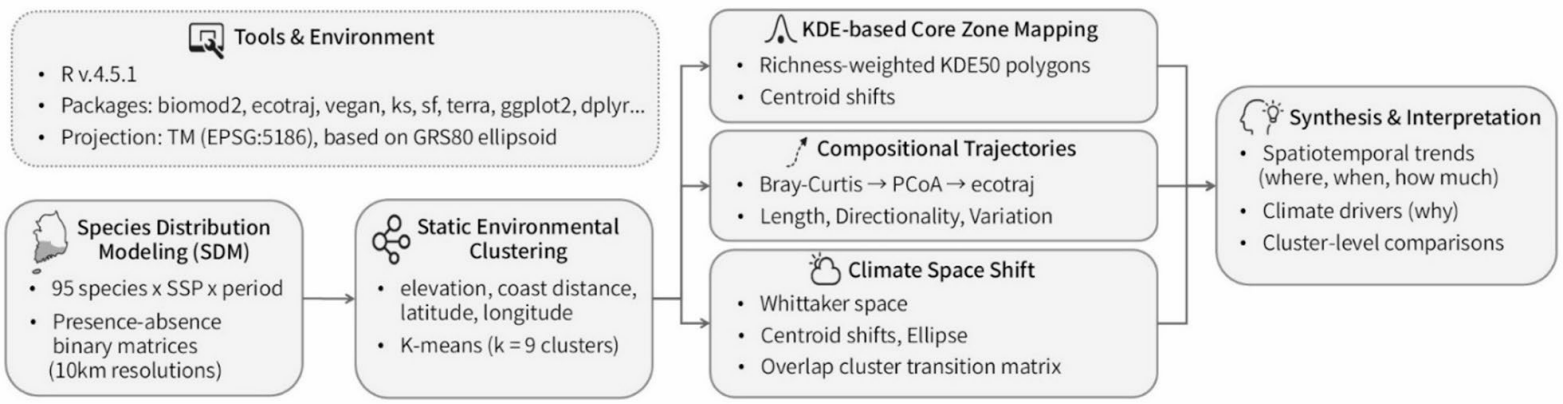
Analytical workflow for climate-driven species distribution analysis. The workflow integrates species distribution modeling (SDM), static environmental clustering, and multiple spatial analyses—including richness-weighted kernel density estimation (KDE), principal coordinates analysis (PCoA), compositional trajectory modeling, and climate space shift detection—culminating in the synthesis and interpretation of species assemblage responses. Core zones were delineated using 50% isopleths derived from KDE (KDE50), representing spatial richness hotspots across climate scenarios. Compositional trajectories were quantified using PCoA outputs processed via the ecotraj framework.

To evaluate the climate-induced spatiotemporal responses of southern plants, we employed a multi-tiered framework integrating SDM, static environmental clustering to delineate spatial ecological zones, and three downstream analyses: (1) mapping core response zones using richness-weighted kernel density estimation (KDE)^31^, (2) ordination-based trajectory analysis of species composition across climate scenarios and time periods^32–34^, and (3) characterization of climatic niche transitions in Whittaker bioclimatic space^26^. This framework links species-level distribution projections with zone-level spatial structure, enabling the assessment of emergent community dynamics within ecologically coherent regions. These sequential components capture species-level projections, emergent zone-level dynamics, and environmental displacements of southern plant assemblages under future climate projections (Fig. 2).

KDE was used to delineate areas of persistent species richness under each scenario and period, providing spatial centroids of static environmental contexts and measuring their geographic shifts. Species compositional changes were examined through ordination-based trajectories, enabling quantification of movement length, directionality, and internal variation^32^. In parallel, Whittaker diagrams constructed from annual temperature and precipitation enabled the identification of potential biome transitions and inter-period envelope overlap.

### Computational environment and common settings

All analyses were performed using R version 4.5.1. Key packages included sf^35^, terra^36^, and ggplot2^37^ for geospatial data handling and visualization; dplyr^38^, tidyr^39^, and purr^40^ for data transformation; biomod2^41^ for SDM; ks^42^ for KDE analysis; vegan^43^ for community dissimilarity and ordination; and ecotraj^32^ for compositional trajectory metrics. Spatial overlays used EPSG:5186-aligned land boundaries, and all grid-based computations were conducted at a uniform resolution of 10 km × 10 km. 30 replicates per cluster were used for trajectory-based sampling per cluster to ensure replicability, and all summary metrics were reported as medians and interquartile ranges unless otherwise noted. Throughout this study, climate scenarios SSP1–2.6, SSP3–7.0, and SSP5–8.5 are abbreviated as 126, 370, and 585, respectively. Similarly, the four time periods are referred to as Current (1980–2010), 1140 (2010–2040), 4170 (2040–2070), and 7100 (2070–2100). All R scripts used for the analyses described in this study are provided in Supplementary Data 2.

### Species occurrence and environmental variables

Based on the list designated by the Korea National Arboretum^44^, occurrence data for 95 southern plant species were compiled from national vegetation surveys and herbarium records (Supplementary Table 1). Sources included government ecosystem inventories and the Korea National Arboretum. To ensure spatial reliability, only species with ≥ 20 confirmed presence records were included. Duplicate or erroneous entries were filtered through quality control in QGIS 3.28, and coordinates were unified using EPSG:5186, consistent with environmental raster layers.

Environmental predictors encompassed climate, soil, and topography. The initial set included 27 bioclimatic variables (CHELSA v2.1), 7 soil parameters, and 4 DEM-derived terrain attributes, all resampled to 250 m resolution and aligned with species data (Supplementary Table 2). To reduce collinearity among environmental predictors, which can adversely affect species distribution model performance^45^, we applied Principal Component Analysis (PCA) to transform correlated variables into orthogonal components. The retained PCA axes represent orthogonal and ecologically interpretable gradients related to soil properties, thermal regimes, precipitation seasonality, and topographic complexity, collectively capturing over 90% of the total variance (Supplementary Table 3).

Future projections followed CHELSA CMIP6 downscaling under SSP1–2.6, SSP3–7.0, and SSP5–8.5. All climate rasters were aggregated to 10 km resolution using mean values, in accordance with the analytical scale of subsequent modeling and spatial analyses.

### SDM construction and binary transformation

Species distribution models were developed using an ensemble learning approach under a presence–pseudo-absence framework. For each species, we generated 1,000 pseudo-absence points across the study area, restricted to locations where all environmental predictors were available and excluding known presence records. To reduce spatial overlap and potential sampling bias, pseudo-absence points were constrained to be at least 10 km away from any presence location. A fixed number of pseudo-absences was used for all species to maintain consistent prevalence and facilitate direct comparability across species within the multi-species ensemble modeling framework.

The ensemble integrated six algorithms: Random Forest (RF), Generalized Additive Models (GAMs), Generalized Boosting Models (GBMs), Classification Tree Analysis (CTA), Artificial Neural Networks (ANNs), and Multivariate Adaptive Regression Splines (MARS). Model performance was evaluated using 5-fold cross-validation implemented within the biomod2 framework. Only models with cross-validated area under the ROC curve (AUC) ≥ 0.7 were retained to ensure acceptable predictive performance. Ensemble projections were then generated by combining retained models using a performance-weighted mean, with weights proportional to their True Skill Statistic (TSS) scores (Supplementary Table 4).

Continuous prediction outputs were converted into binary presence–absence maps using the maximum TSS (True Skill Statistic) thresholding rule. The resulting binary rasters were aggregated into a species-by-grid matrix (10 km resolution) used in downstream community-level and spatial analyses.

The species distribution modeling workflow followed standardized protocols described in a previous study^11^, including pseudo-absence generation, model calibration, cross-validation, and ensemble prediction.

### Spatial clustering based on static environmental variables

To delineate scenario-invariant spatial ecological zones, we applied unsupervised clustering to four static spatial variables: elevation, distance to coast, latitude, and longitude. Each variable represents a fundamental and largely time-invariant geographic constraint shaping plant distributions in South Korea. Elevation captures long-term thermal and orographic limitations, distance to coast reflects persistent maritime influence on temperature and moisture regimes, and latitude and longitude summarize broad biogeographic gradients and spatial context across the peninsula. By restricting clustering to these static variables, we established a stable spatial framework within which dynamic climatic and compositional changes could be evaluated consistently across multiple climate scenarios.

All variables were harmonized to the same analytical resolution used for climate predictors. Elevation was derived from the Shuttle Radar Topography Mission (SRTM) 3 arc-second (~ 90 m) global digital elevation model provided by NASA. Distance to coast was calculated as the Euclidean distance from each raster grid cell to the nearest shoreline, with the coastline represented as a vector layer extracted from the GADM version 4.1 national boundary dataset. For a small number of nearshore grid cells, distance to coast values were missing due to partial overlap between land–sea masks during resampling. These missing values were interpolated solely to ensure spatial continuity using a generalized additive model (GAM) with distance to coast as a smooth predictor, employing a Gaussian error distribution with an identity link and default smoothing settings, as this step served only as a boundary correction rather than for inferential purposes^46^. Geographic coordinates were initially processed in the projected coordinate system EPSG:5186 to ensure accurate distance calculations and spatial operations. The data were subsequently transformed to EPSG:4326 solely to extract latitude and longitude values for use as static spatial predictors and for interpretability, rather than for distance-based analyses or clustering. All variables were standardized via z-transformation prior to clustering. K-means clustering was applied, and the number of clusters (k = 9) was selected by constraining candidate solutions to k ≥ 6 to ensure sufficient ecological resolution, within which silhouette values formed a broad plateau rather than a sharp maximum^47^. Expert-based evaluation was used as a secondary criterion to assess ecological interpretability and spatial coherence (Supplementary Fig. 1), based on whether clusters exhibited contiguous spatial patterns and aligned with major elevational and coastal gradients relevant to southern plant distributions. Although k-means clustering does not explicitly account for spatial autocorrelation, our objective was to delineate ecologically interpretable and scenario-invariant spatial zones based on static geographic gradients, rather than to optimize spatial contiguity. The final clustering yielded scenario-invariant spatial units that were subsequently used as a common spatial framework for downstream analyses, including the identification of core response zones, compositional trajectory analysis, and climatic niche transitions of southern plant assemblages under climate change.

### Estimation of climate response zones via richness-weighted kernel density estimation

To identify spatially coherent core response zones representing areas of persistent occurrence of southern plant assemblages under each climate scenario and time period, we applied richness-weighted kernel density estimation (KDE). This approach emphasizes regions where multiple southern plant species are projected to co-occur, thereby delineating conservative community-level core response zones rather than complete species ranges. KDE results were interpreted in conjunction with scenario agreement and downstream compositional trajectory analyses to reduce sensitivity to uncertainty inherent in SDM-based richness estimates. KDE was conducted using grid cells with non-zero predicted species richness, with richness values serving as replication weights. Adaptive bandwidths were estimated using a data-driven plug-in selector that optimizes the kernel bandwidth matrix based on the empirical distribution of the input points^42^. Based on these bandwidths, 50% isopleth contours (KDE50) were extracted to delineate core response zones^48^. The resulting polygons were clipped to a unified terrestrial boundary to exclude marine areas.

To compare spatiotemporal dynamics among species clusters, KDE50 regions were summarized at the cluster level. For each cluster and time period, KDE50 polygons were spatially merged by dissolving all KDE50 regions belonging to the same cluster into a single composite polygon. The geographic position of each merged KDE50 region was represented by its geometric centroid, from which longitude and latitude were extracted. In contrast, elevation and distance to coast were summarized across the full extent of each merged KDE50 region using area-weighted raster extraction, yielding mean, median, and interquartile range values that capture within-region environmental heterogeneity^49^.

Changes in these attributes between the current period and late-century projections (SSP5–8.5, 2100) were subsequently quantified for each cluster, allowing assessment of both the magnitude and direction of geographic displacement, including latitudinal shifts, inland movement, and elevational transitions.

### Trajectory analysis of species compositional change

Bray–Curtis dissimilarities derived from binary species presence–absence matrices were projected into ordination space to trace compositional trajectories across four time periods^50^. For each climate scenario, matrices were constructed using grid cells with complete species predictions across all four time points (Current, 1140, 4170, and 7100).

To ensure comparable sample sizes across clusters, reduce bias associated with unequal cluster extents, and maintain computational tractability for repeated trajectory estimation, we employed a repeated subsampling strategy. For each of the nine environmental clusters, 20 grid cells were randomly sampled without replacement, a number chosen to balance spatial representativeness within KDE50 regions and computational efficiency while allowing repeated estimation of trajectory metrics. This procedure was independently repeated 30 times to account for stochastic variation arising from random selection. Trajectory metrics were calculated for each replicate, and final estimates were summarized using median values and interquartile ranges, providing robust measures of cluster-level compositional change.

Ordination was performed using principal coordinate analysis (PCoA) to generate low-dimensional projections of community dissimilarity^33^. For each sampled grid cell, a compositional trajectory was defined in ordination space across successive time periods. Trajectories were constructed by sequentially connecting the ordination scores of each grid cell across time. Three complementary trajectory metrics were calculated to characterize different aspects of compositional change. Trajectory length was defined as the cumulative Euclidean distance between successive time points in ordination space, representing the overall magnitude of compositional change through time. Directionality was calculated as the ratio of net displacement between the initial and final positions to the total trajectory length, with values approaching one indicating straight, unidirectional change and lower values indicating more circuitous trajectories. Internal variation was quantified as the standard deviation of turning angles between successive trajectory segments, reflecting the degree of irregularity or nonlinearity in the compositional path. Together, these metrics capture the magnitude, coherence, and internal variability of compositional trajectories under climate change^34^.

To evaluate whether compositional shifts followed underlying environmental gradients, we applied vector fitting using standardized environmental variables (elevation, distance to coast, mean annual temperature, and precipitation seasonality). For each cluster–scenario replicate, directional alignment was quantified as the cosine of the angular deviation (cos Δθ) between the trajectory vector and each environmental vector, with values ranging from − 1 (opposition) to 1 (perfect alignment). To incorporate both the magnitude and direction of compositional change, we calculated scaled alignment explicitly as the product of trajectory length (L) and cos Δθ (L × cos Δθ). In this formulation, the sign of scaled alignment indicates whether compositional change proceeds along or against an environmental gradient, while its absolute value reflects the strength of environmentally aligned compositional change. For each replicate, the environmental variable with the highest absolute scaled alignment was interpreted as the dominant driver of community change.

We decomposed compositional turnover across three temporal intervals (Current–1140, 1140–4170, and 4170–7100) by calculating pairwise Bray–Curtis dissimilarities within each replicate, allowing turnover magnitude to be quantified separately for early- and late-century transitions and enabling temporal comparison of compositional change dynamics.

Finally, to statistically assess differences in community composition among environmental clusters and time periods, we conducted permutational multivariate analysis of variance (PERMANOVA) based on Bray–Curtis dissimilarities. A global PERMANOVA model was fitted with cluster identity and time period as explanatory factors using 9,999 permutations. Effect sizes were quantified using R² values to compare the relative contribution of each factor to overall compositional variation. To evaluate whether observed differences were influenced by changes in within-group dispersion, we additionally conducted a permutational analysis of multivariate dispersions (PERMDISP).

### Climate space shifts of environmental clusters on the Whittaker diagram

To assess climate-driven shifts in southern plant assemblages, each 10 km grid cell was projected onto a two-dimensional Whittaker climate space defined by mean annual temperature (MAT) and annual precipitation (MAP)^52^. Cluster identities, previously derived from environmental classifications, were preserved, allowing temporal comparison of climate space occupancy.

For each cluster, climatic centroids were calculated as the average MAT and MAP across all assigned grid cells for both current and future periods (2070–2100 under SSP5–8.5). Internal climatic dispersion was summarized by fitting 50% and 95% confidence ellipses based on multivariate normal approximation^53^. Changes in ellipse areas between time periods were then computed (ΔArea), providing an estimate of expansion or contraction in climate occupancy and a structural indicator of niche breadth dynamics^54^.

To assess temporal continuity of cluster-specific climate spaces, we computed two polygon-based overlap metrics: (1) the Jaccard index, representing the proportion of intersected area relative to the union of ellipses; and (2) the minimum-based overlap, defined as the intersected area over the smaller ellipse. These metrics were calculated via spatial intersection of rasterized ellipses.

Beyond aggregate climate space changes, we examined transitions of individual grid cells between clusters in MAT–MAP space. Each cell’s current and future cluster labels were compared to construct a 9 × 9 transition matrix. Each entry, denoted as P(F|C), represents the proportion of area currently classified in cluster C that transitions to future cluster F^55^. These proportions were derived from spatial overlays and summarized in a proportional heatmap to highlight dominant inter-cluster shifts within climate space.

A digitized Whittaker biome reference was overlaid for interpretative context, allowing each cluster’s climate trajectory to be viewed within a broader biome classification framework. This analysis complements spatial metrics by elucidating the bioclimatic displacement and restructuring of southern plant distributions, assessed through changes in both central tendency and dispersion within climate space.

## Results

### Environmental delineation and spatial profiles of static clusters

**Fig. 3:**
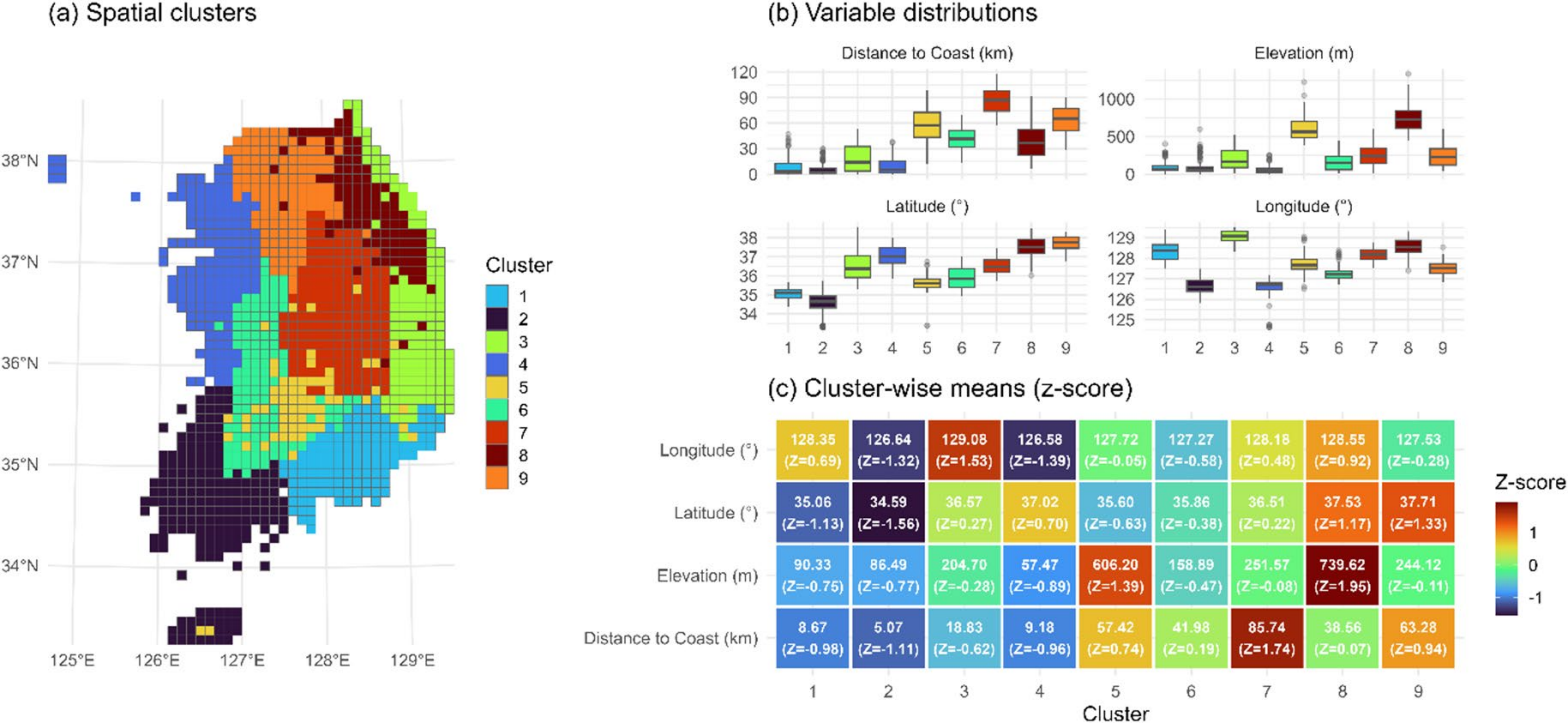
Spatial clustering and environmental diagnosis of sample grid cells. Panel (**a**) shows the spatial distribution of nine environmental clusters across the study area. Panel (**b**) presents boxplots of key environmental variables by cluster, while (**c**) summarizes the standardized means (z-scores) of the same variables. These visualizations provide baseline environmental context for subsequent species composition and trajectory analyses. All panels and drawings were generated by the authors using R version 4.5.1 (https://www.R-project.org/) with the ‘ggplot2’ (https://ggplot2.tidyverse.org) and ‘sf’ (https://r-spatial.github.io/sf/) packages.

Environmental clustering based on static variables resulted in nine distinct spatial clusters, each characterized by unique combinations of latitude, longitude, elevation, and distance to the coastline (Supplementary Table 5). These clusters exhibited clear geographical gradients and environmental stratification across the Korean Peninsula (Fig. 3).

In terms of latitudinal distribution, Clusters 8 and 9 represented the northernmost zones (z = + 1.11 and + 1.25, respectively), while Cluster 2 occupied the southernmost region (z = − 1.38), particularly along the southern coastline. Longitudinally, Cluster 3 showed the most eastern distribution (z = + 1.48), whereas Clusters 2 and 4 were centered in the western lowland zones (z = − 1.11 and − 1.17, respectively).

With respect to elevation, Cluster 8 exhibited the highest mean elevation (739.6 m, z = + 2.18), followed by Cluster 5 (606.2 m, z = + 1.61), both corresponding to inland mountainous areas. In contrast, Clusters 1, 2, and 4 had the lowest mean elevations (< 100 m, z < − 0.6), and were mainly distributed along low-elevation coastal plains.

Distance to the coast further distinguished clusters by their degree of inlandness. Cluster 7 had the greatest mean distance from the sea (85.7 km, z = + 1.60), followed by Cluster 9 (63.3 km, z = + 0.90), both reflecting deeply inland locations. Conversely, Clusters 2 and 1 were strongly coastal (5.1 km and 8.7 km, respectively; z < − 0.8), indicating immediate proximity to marine environments.

When integrating these variables, several notable environmental identities emerged. Cluster 8 represented the highest elevation and northernmost mountainous region, combining high latitude, eastern longitude, and considerable inland distance. Cluster 2, in contrast, marked the southern coastal lowland, with the lowest latitude, elevation, and sea distance. Cluster 5 was distinguished as a central highland cluster, while Cluster 7 formed the deepest inland region, being furthest from the coast despite moderate elevation. Clusters 1 and 4 represented coastal lowland zones on the southeast and southwest, respectively, while Cluster 6 aligned closely with national environmental averages across all variables.

### Dynamics of richness-weighted 50% kernel density hotspot regions and cluster-level redistribution

Across the study periods, richness-weighted 50% kernel density (KDE50) hotspot regions exhibited pronounced temporal reorganization, indicating a progressive redistribution of core areas supporting southern plant assemblages under future climate conditions. Summary metrics (Supplementary Table 5) show that mean total KDE50 area increased from 47,184.7 km² in the current period (1980–2010) to 49,652.4 km² in the late-century projection (2070–2100), while the average cluster-level median polygon area expanded from 287.4 km² to 1,438.4 km². In contrast, the number of discrete KDE50 polygons declined from 10.0 to 7.0 on average, and the variability (standard deviation) of both total and median area increased over time.

**Fig. 4:**
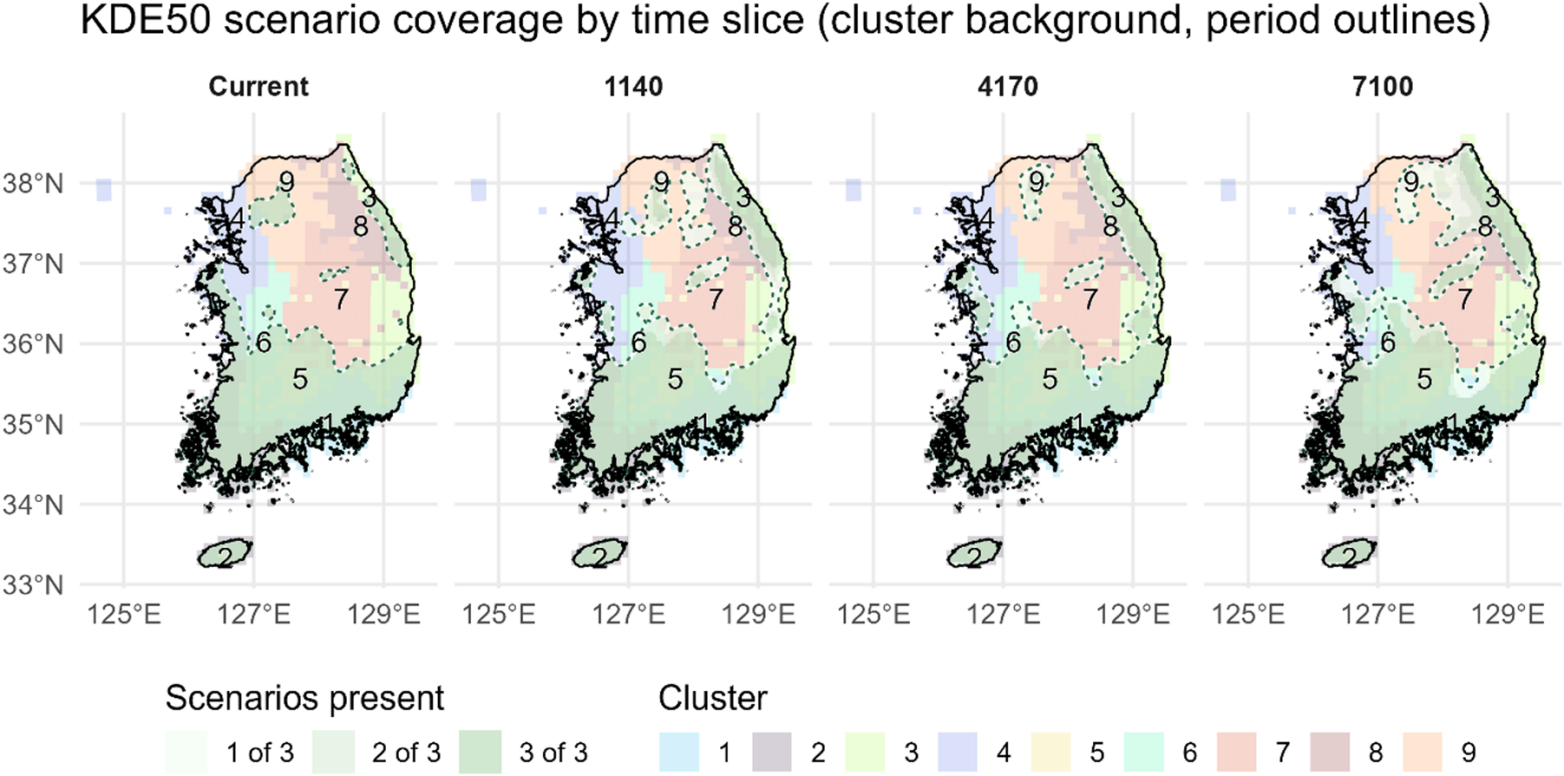
Temporal 50% KDE occupancy and scenario agreement. Each panel displays the dominant static environmental cluster per grid cell (shown as categorical background colors) and the number of climate scenarios in which the cell falls within the 50% kernel density area of that cluster (overlaid using a sequential color scale to represent scenario agreement). For example, “3 of 3” indicates inclusion in all three scenarios, reflecting high spatial consensus. The combined visualization allows simultaneous interpretation of static spatial structure and cross-scenario consistency of KDE50 core zones. Time periods are abbreviated as Current (1980–2010), 1140 (2010–2040), 4170 (2040–2070), and 7100 (2070–2100). The maps were generated by the authors using R version 4.5.1 (https://www.R-project.org/) with ‘ggplot2’ (https://ggplot2.tidyverse.org), ‘sf’ (https://r-spatial.github.io/sf/), and ‘terra’ (https://rspatial.github.io/terra/) packages.

Spatially, KDE50 core zones show a progressive redistribution toward higher latitudes and elevations, with some clusters also exhibiting inland displacement (Fig. 4). These directional shifts are quantitatively supported by increases in centroid latitude, northern range limits, mean elevation, and distance to coast across successive time periods (Supplementary Table 6). Scenario agreement among hotspot cells tends to increase across central and southern inland areas, while peripheral coastal zones display greater spatial heterogeneity and lower inter-scenario overlap.

Before turning to cluster-specific centroid trajectories, it is worth noting that the KDE50 centroid for all southern plant species combined shifted northward (~ 0.2° latitude), upslope (~ + 70 m), and slightly inland (~ + 3 km) between the current period and 7100 under SSP5–8.5, indicating an overall displacement toward cooler, higher, and more interior environments(Supplementary Table 7). Cluster-level patterns further illustrate these heterogeneous dynamics. Clusters 1, 2, and 3 maintained persistent representation within KDE50 areas throughout all periods, serving as stable components of core richness zones under both current and projected conditions. Cluster 4, typically located outside hotspot boundaries in the baseline, gradually expanded its KDE50 coverage. Clusters 5 and 6 contributed to increased median polygon sizes, particularly in mid- and late-century intervals. Notably, clusters 7, 8, and 9 exhibited the most substantial gains in KDE50 representation, transitioning from initially fragmented patches into larger, spatially cohesive hotspot areas in future projections.

**Fig. 5:**
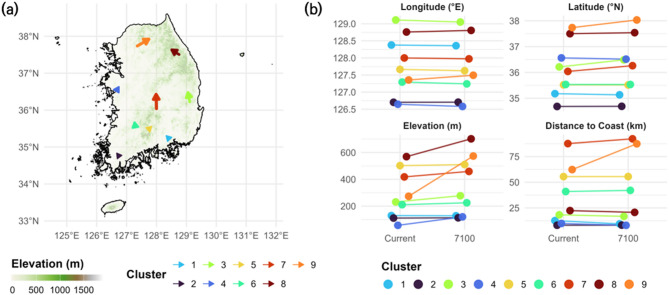
Directional shift of cluster centroids and associated environmental changes. Panel (**a**) depicts arrows indicating the movement of KDE50% centroids for each cluster from the current period to the future (2070–2100, integrated across SSP1–2.6, 3–7.0, and 5–8.5). Arrow length is proportional to the spatial shift distance (km). Panel (**b**) summarizes changes in the mean longitude (°), latitude (°), elevation (m), and coastal distance (km) of grid cells within each cluster. Line colors differentiate clusters, while arrow direction and length reflect the magnitude and orientation of environmental shifts. The maps and plots were generated by the authors using R version 4.5.1 (https://www.R-project.org/) with ‘ggplot2’ (https://ggplot2.tidyverse.org), ‘sf’ (https://r-spatial.github.io/sf/), and ‘terra’ (https://rspatial.github.io/terra/) packages.

Spatial reorganization of KDE50 core zones is further supported by cluster-level centroid movement and associated environmental attributes (Fig. 5). Under the 7100 SSP5–8.5 scenario, most clusters show displacement of their KDE50 centroids toward higher latitudes and elevations. Latitude increased in clusters 6, 7, and 9, while elevation rose most clearly in clusters 8 and 9. In terms of coastal distance, clusters 7 and 8 showed consistent increases in mean distance from the coastline. Longitudinal change was less consistent and varied across clusters without a dominant directional trend. These results indicate that the environmental conditions of KDE50 centroids—defined by latitude, elevation, and coastal proximity—have shifted over time, with the degree and direction of change varying across clusters.

### Climate-driven compositional trajectories and environmental alignment

**Fig. 6:**
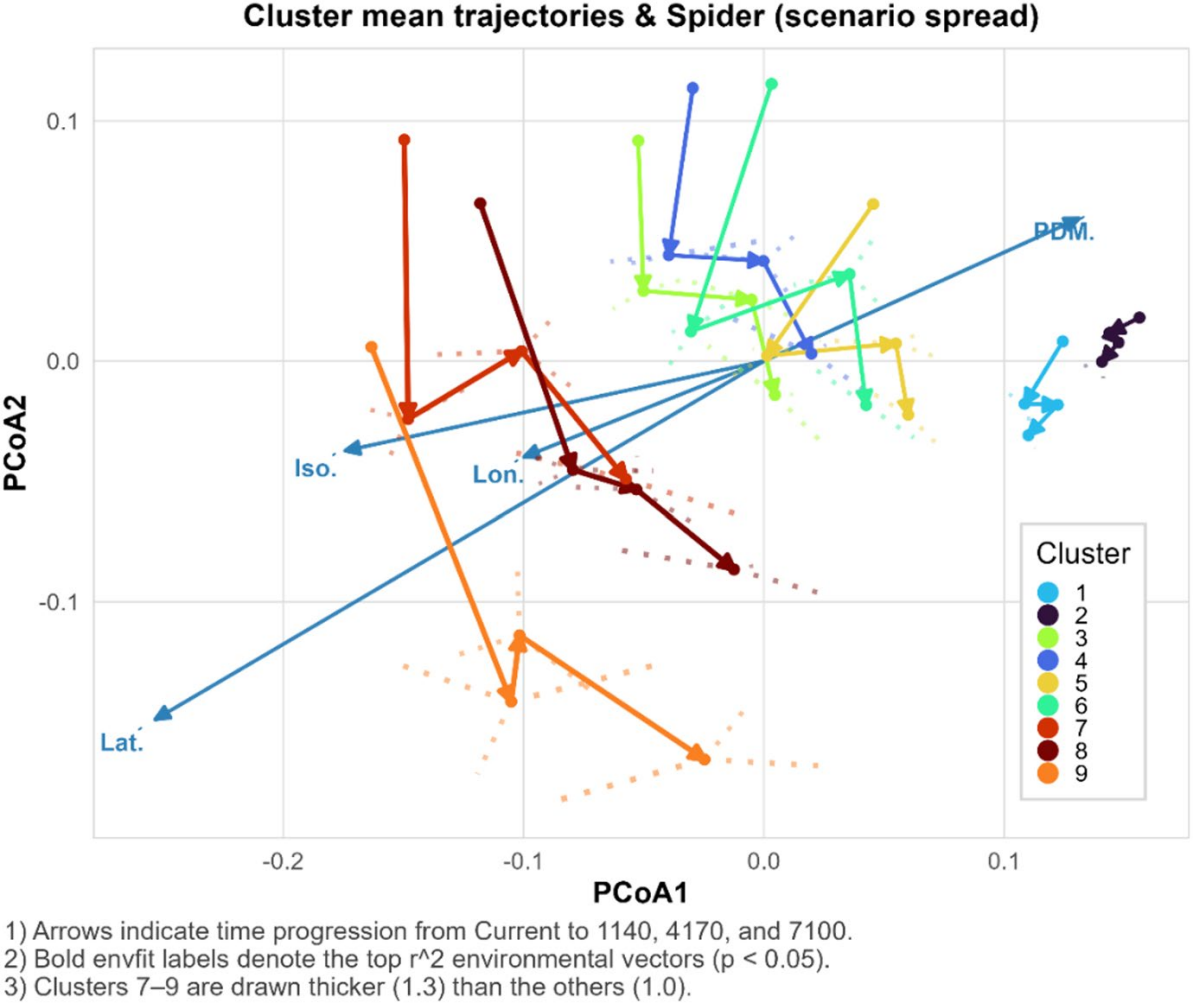
Temporal trajectories of species clusters in PCoA space under climate change scenarios. PCoA based on Bray–Curtis dissimilarities with Lingoes correction was used to visualize temporal shifts in species composition centroids across periods. Arrows represent directional change from present to future (1140, 4170, 7100), and spider lines indicate within-period variation across scenarios. Dark blue envfit vectors denote environmental variables with significant explanatory power (r², *p* < 0.05), including Latitude (Lat.), Longitude (Lon.), Isothermality (Iso.), and Precipitation of the Driest Month (PDM.). Trajectories for clusters 7–9 are displayed with 1.3× thicker lines to highlight higher ecological shift potential. Because ordinations from multiple subsets and replicates are combined on a single reference plane, axis variance percentages are not shown. The plots were generated by the authors using R version 4.5.1 (https://www.R-project.org/) with ‘ggplot2’ (https://ggplot2.tidyverse.org) and ‘ecotraj’ (https://cran.r-project.org/package=ecotraj) packages.

The PCoA ordination revealed consistent temporal shifts in species composition across climate change scenarios, illustrating how plant communities move through compositional space as climatic conditions change. Most clusters exhibited progressive movement from the present toward future periods (1140, 4170, and 7100), with Clusters 7–9 showing the longest and most directional trajectories (Fig. 6). In particular, trajectories of Clusters 1 and 2 were primarily aligned with the latitude vector, indicating northward compositional shifts, whereas Clusters 7–9 showed strong directional movement aligned with isothermality but in the opposite direction, suggesting pronounced community restructuring rather than simple latitudinal tracking (Fig. 6).

Beyond these mean directional shifts, within-period variation across scenarios increased over time, as illustrated by spider plots. This increase was especially pronounced in environmentally extreme clusters, indicating that climate change not only displaced average community composition but also amplified internal heterogeneity among scenario-specific projections. Such divergence suggests growing uncertainty and diversification of compositional responses within clusters under more extreme future conditions.

Consistent with these patterns, fitting of environmental vectors (envfit) identified four variables—latitude, longitude, isothermality, and precipitation of the driest month—as significantly correlated with compositional gradients (r² > 0.3, *p* < 0.05). The orientation of these vectors broadly matched the directions of cluster trajectories in ordination space, indicating that both geographic position and climatic extremes jointly structured community dynamics under future climate scenarios.

Global PERMANOVA supported these patterns, showing that cluster identity (R² = 0.364, F = 57.79, *p* < 0.001) accounted for over four times more variation than period (R² = 0.083; Supplementary Table 8). All nine clusters exhibited significant temporal changes (*p* < 0.001), with Clusters 8 and 9 displaying the strongest responses (R² = 0.333 and 0.386). In contrast, Clusters 1–4 showed minimal compositional shifts (R² < 0.14), indicating temporal stability.

PERMDISP analysis revealed increased multivariate dispersion over time (F = 41.16, *p* = 0.001), consistent with spider plots and suggesting that climate change affected not only mean composition but also within-cluster variability.

### Cluster-specific Trajectory Metrics and Variance Decomposition

**Fig. 7:**
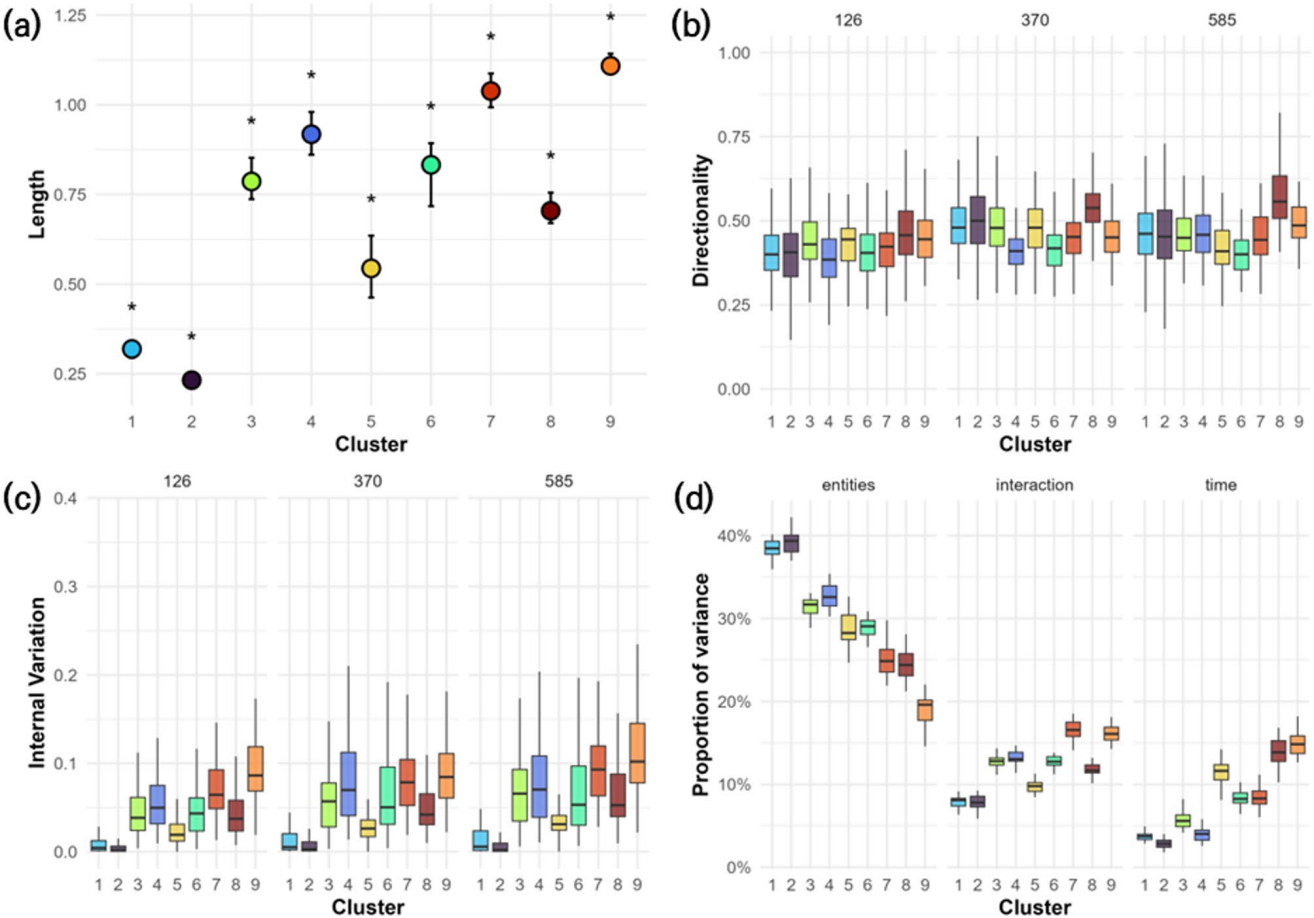
Cluster trajectory characteristics and variance contributions under climate scenarios. This figure illustrates the ecological responses of nine clusters under three climate scenarios (126 = SSP1–2.6, 370 = SSP3–7.0, 585 = SSP5–8.5), using four trajectory metrics across time. Panel (**a**) shows the trajectory length, quantified as the centroid movement over time. Panel (**b**) presents trajectory directionality, reflecting the consistency and linearity of cluster movements across time periods. Panel (**c**) visualizes within-cluster variation in species composition among grid cells, and panel (**d**) displays the proportion of variance in species composition explained by cluster identity (entities), time period (time), and their interaction (interaction), highlighting the dominant role of spatial clustering in structuring ecological trajectories. Asterisks in panel (a) denote statistically significant differences across scenarios within each cluster (Tukey’s HSD, *p* < 0.05), while panels (b–d) are presented for comparative interpretation without statistical annotations. The plots were generated by the authors using R version 4.5.1 (https://www.R-project.org/) with the ‘ggplot2’ (https://ggplot2.tidyverse.org) package.

Cluster-level trajectory metrics revealed clear heterogeneity in compositional responses among spatial clusters, indicating contrasting degrees of stability and reorganization across the landscape (Fig. 7).Trajectory length differed significantly among clusters (Tukey’s HSD, *p* < 0.05), with Clusters 7–9 showing the greatest shifts and Clusters 1–2 the shortest. These patterns mirrored earlier PERMANOVA results (Fig. 7a). Trajectory directionality was moderate across clusters (median ≈ 0.5), reflecting a mix of consistent and stochastic changes. Clusters 7–9 again showed higher directionality, implying more coordinated shifts under climate pressure (Fig. 7b). Internal variation was lowest in Clusters 1–2 and highest in Clusters 7–9, suggesting that compositional divergence among grid cells increased in topographically or climatically complex regions (Fig. 7c). Variance partitioning further emphasized the dominant role of spatial clustering, with cluster identity explaining over 30–40% of total variance. Temporal effects were most pronounced in Clusters 7–9, while the cluster × time interaction term contributed non-negligibly in several clusters, suggesting differential sensitivity to climate change within spatial units (Fig. 7d).

Alignment analysis revealed that Clusters 1 and 2 tracked latitudinal gradients (cos Δθ > 0.85) with modest displacement, whereas Clusters 3–6 showed oblique or misaligned shifts (cos Δθ = − 0.45 to 0.28), indicating complex responses. Clusters 7–9 moved against the isothermality gradient (cos Δθ = − 0.54 to − 0.65), reflecting nonlinear responses possibly linked to biome instability (Supplementary Table 9).

Collectively, these results underscore that community trajectories are highly cluster-specific—in both magnitude and direction—and strongly modulated by underlying environmental gradients.

### Climate space reorganization and biome transitions of plant community clusters

**Fig. 8:**
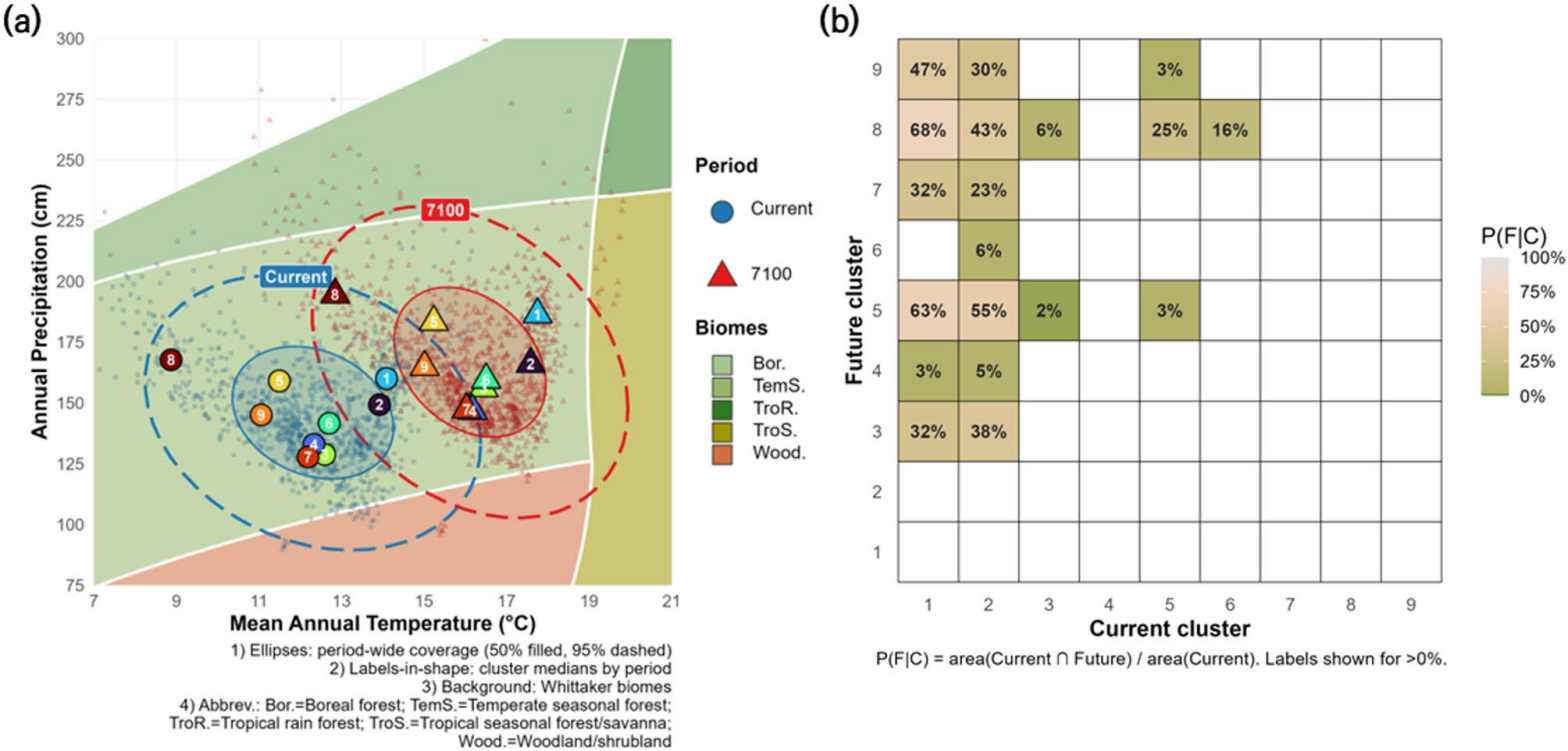
Cluster shifts and overlap within climate space. Panel (**a**) plots cell-wise mean annual temperature (°C) and precipitation (mm) for the present (blue circles) and future (2070–2100, red triangles) over a Whittaker biome diagram. Cluster centroids are labeled by number, and 50% and 95% confidence ellipses represent the overall distribution range of the study area’s climate space. Panel (**b**) shows a heatmap of the overlap proportion between current clusters (C) and future clusters (F) in climate space. For instance, a value of 68% in cell (1,8) indicates that 68% of the climate space occupied by current cluster 1 overlaps with that of future cluster 8. This highlights that cluster identities may shift over time and that inter-cluster transitions are possible under future climate conditions. The plots were generated by the authors using R version 4.5.1 (https://www.R-project.org/). The background Whittaker biome diagram was created using the ‘plotbiome’ R package (https://github.com/valentinitnelav/plotbiome).

This subsection synthesizes multiple lines of evidence to describe how plant community clusters reorganize within climate space under future scenarios. Patterns of climate space reconfiguration were derived from density hotspots, centroid movement, cluster identity changes, and biome-level transitions within the Whittaker framework. In the MAT–MAP climate space (Fig. 8a), current (blue) and future (red) cluster centroids exhibited a clear directional shift toward warmer and wetter conditions. The 95% confidence ellipse expanded by 4.5% (from 702.05 to 733.72 °C·cm), reflecting a modest increase in climate space (Supplementary Table 10). However, low overlap metrics (Jaccard: 0.183; minimum-based: 0.317) suggest that this expansion was accompanied by significant displacement, rather than simple broadening of existing conditions. Reflecting this shift, Biome classification based on the 95% ellipses indicated a decline in temperate seasonal forest coverage (from 90.8% to 84.3%), accompanied by the emergence of tropical seasonal forest (7.0%) and temperate rain forest (1.1%) in future conditions—biomes absent in the current period. These patterns suggest not only within-biome shifts but also functional transitions into new climatic regimes.

A heatmap of cluster-level overlaps (Fig. 8b) revealed asymmetric inter-cluster correspondences. For example, 68% of the climate space occupied by current Cluster 1 overlaps with that of future Cluster 8, while the reverse overlap is minimal. This implies a northward shift in climate niches, particularly for low-latitude clusters. This pattern underscores the directional nature of climate-driven redistribution, leading to partial disjunction between current and future community configurations. These tendencies are further supported by the analysis of cluster-specific environmental characteristics (Supplementary Table 4). Clusters 1 and 2 are defined by southern lowland environments, whereas clusters 7 to 9 occupy higher latitudes and elevations, reflecting a latitudinal and elevational gradient across the study region (Fig. 3). Standardized z-scores for elevation and sea distance align consistently with this spatial ordering, reinforcing the interpretation that future displacements involve both niche drift and systematic reordering along latitude and elevation axes. Notably, clusters 5 and 8, which exhibit high elevations (606.2 m and 739.6 m) and substantial sea distances, may function as indicating their potential as future climate refugia under climate change scenarios.

## Discussion

This study offers a spatially explicit, multi-scenario framework for assessing community-level responses of southern Korean vascular plant assemblages under climate change. In line with the objectives outlined in the Introduction, we discuss our findings in terms of temporal compositional reorganization, spatial differentiation of stable versus unstable response zones, and climate-space displacement of community clusters. By combining SDM, spatial clustering, KDE, ordination-based trajectory metrics, and environmental alignment diagnostics, we captured the heterogeneous dynamics of compositional reorganization across both space and time. Specifically, SDM describes potential shifts in suitable conditions for individual species, spatial clustering provides a stable geographic context for comparison, KDE identifies zones of persistent assemblage occurrence, and trajectory-based analyses quantify how, when, and along which environmental gradients community composition changes. Rather than assuming homogeneous range shifts or uniform community responses, our results reveal highly differentiated patterns among spatial clusters, contingent on climatic pathways and landscape context^12–14^. These findings reinforce our hypothesis that climate-driven vegetation responses are spatially structured and that integrating directional and variability metrics provides improved interpretability for conservation and restoration planning.

We classified the identified clusters into three interpretative groups—stable, transitional, and transformational—based on the magnitude of compositional shifts, alignment with environmental gradients, and scenario agreement. Stable clusters (e.g., Clusters 1–3) showed minimal compositional shifts and strong directional alignment, suggesting persistence cores that may function as long-term climate refugia^11^. Transformational clusters (e.g., Clusters 7–9) exhibited large trajectory lengths and weak or negative alignment with climatic gradients, indicating high reorganization and uncertainty, and thus may facilitate non-analog community reassembly^21,57,58^. Transitional clusters (e.g., Clusters 4–6) displayed intermediate dynamics and may serve as buffer zones relevant to adaptive landscape connectivity. To illustrate the ecological meaning of these response types, stable clusters in southern coastal and insular regions may preferentially retain evergreen broadleaf forest assemblages and coastal/insular endemic species, whereas transitional clusters may increasingly represent zones of overlap where evergreen broadleaf and subtropical-affiliated taxa expand northward into temperate deciduous communities, potentially intensifying biotic interactions. In transformational clusters, projected reassembly may lead to new co-occurrence contexts between southern-affiliated taxa and boreal-affiliated communities, raising uncertainty for persistence of boreal endemic refugial assemblages. A key analytical advantage of our framework lies in linking compositional trajectories with environmental gradients using directional alignment metrics, enabling interpretation of whether community change proceeds along, against, or independently of dominant climatic gradients^32^. Directional misalignment may also reflect constraints imposed by static environmental structures and localized buffering (e.g., topography, marine influence, cold-air pooling), as well as biotic processes such as compositional inertia or priority effects^50,59–61^. Recognizing these mediating mechanisms is essential when interpreting misalignment as either ecological risk or an alternative stabilization pathway under environmental complexity^62,63^.

Beyond these ecological interpretations, our framework introduces methodological innovations with direct policy relevance. In contrast to studies focused mainly on static climate envelopes, range centroids, or richness counts, our approach integrates KDE-based hotspot mapping with trajectory metrics. This combination enables simultaneous detection of spatial cores of persistence and directional dynamics of change, offering a more dynamic basis for forecasting biodiversity responses^57,64^. First, by modeling compositional responses at the community level, we better capture functional and structural ecosystem shifts^12,13^. Second, the use of KDE50 polygons identifies spatial cores of stability, enabling geographic prioritization that integrates variability and scenario uncertainty^65^. Third, the trajectory analysis based on Bray–Curtis dissimilarity captures the path of ecological reorganization, rather than merely its endpoints^32,66^. Unlike traditional trajectory studies that rely on empirical plot sequences or static climate binning, our approach mitigates key scenario-based limitations. We ensure full temporal coverage across fixed spatial units by filtering for consistently predicted grid cells, and apply repeated sampling to address internal variability. The use of L × cos Δθ enables simultaneous quantification of compositional change magnitude and its directional alignment with climate gradients—an interpretive advance over metrics that assess only path length. This integration of dynamic directionality and environmental coupling strengthens the explanatory value of trajectory-based assessments under future scenarios. While KDE-based mapping highlights spatial cores of compositional resilience, trajectory metrics complement this by providing dynamic insights into the magnitude and direction of temporal change. Together, they offer a dual perspective (spatial stability and directional movement), enabling a more comprehensive interpretation of vegetation responses across space and time. This dual-framework allows us to distinguish not only where ecological persistence is likely, but also how and in what direction compositional changes are unfolding. Finally, the multi-scenario framework spanning SSP1–2.6, 3–7.0, and 5–8.5 expands applicability to policy environments with varying assumptions about socio-economic trajectories. These technical enhancements bridge predictive ecology and spatial planning in a reproducible and scalable manner^67^.

From a policy perspective, this study aligns closely with recent global conservation frameworks such as the Kunming–Montreal Global Biodiversity Framework (GBF), which emphasize spatially coherent and climate-resilient biodiversity strategies (Supplementary Table 11). First, clusters exhibiting high compositional stability and strong climatic alignment—such as the Southern Coastal Lowland, Southern Maritime Zone, and Eastern Inland Lowland (Clusters 1–3)—can be interpreted as potential climate refugia^68^. These areas may be prioritized as core zones for long-term protection, strengthened monitoring networks, or strict conservation reserves. Second, transitional clusters characterized by moderate compositional shifts and partial environmental alignment—namely the Western Coastal Plain, Central Highland Zone, and Central Inland Transition (Clusters 4–6)—are likely to function as ecological corridors or adaptation pathways, facilitating species movement and buffering climate-driven range shifts^60,69^. These zones are particularly relevant for designing climate-smart connectivity strategies across heterogeneous landscapes. Third, clusters showing pronounced instability and directional incongruence—such as the Eastern Inland Upland, Northern Montane Zone, and Northern Inland Upland (Clusters 7–9)—represent high-uncertainty regions of ecological reorganization, potentially involving nonlinear or threshold-like responses to climatic forcing^11,70^. These regions warrant proactive monitoring, experimental restoration, and adaptive management frameworks tailored to uncertainty. The stability maps and directional indicators developed herein can inform region-specific conservation zoning, adaptive restoration strategies, and national biodiversity reporting under the GBF framework. By integrating ecological coherence and climate responses across spatial units, this framework supports more robust decision-making in the face of environmental volatility and long-term change. In practice, these recommendations may be operationalized in Korea by integrating cluster-level stability and alignment metrics into existing protected-area planning, long-term vegetation monitoring schemes, and adaptive management plans implemented at national and regional scales. To enhance practical applicability, we additionally illustrate how response types intersect with Korea’s national park network by overlaying cluster distributions with national park boundaries (Supplementary Fig. 2), providing a park-scale decision-support context for climate-adaptive monitoring and spatial prioritization.

Importantly, this study also contributes to the growing body of work emphasizing dynamic rather than static conservation paradigms^71,72^. Our results suggest that conventional, fixed protected area networks may not be sufficient to sustain biodiversity in the face of ongoing and anticipated climatic shifts. Instead, there is a critical need for flexible and updateable conservation zoning, informed by real-time ecological monitoring and scenario-based forecasts^4,73^. Because our method is reproducible and relatively computationally efficient, it can be embedded into routine national monitoring systems or regional restoration planning processes.

Nonetheless, several methodological limitations should be acknowledged. A primary constraint arises from assumptions inherent to species distribution models (SDMs), which implicitly assume quasi-equilibrium between current species distributions and contemporary climate conditions. Under rapid climate change, SDM projections therefore represent potential climatic suitability rather than realized future distributions, as they do not explicitly account for dispersal limitation, biotic interactions, or historical land-use legacies. This limitation implies that our results should be interpreted in terms of relative spatial and compositional reorganization rather than absolute predictions of species presence.

In addition, rare species or microsite-specific responses may be underrepresented, potentially influencing community-level inferences^74,75^. The cosine-based alignment metric further assumes linear responses to environmental gradients and may oversimplify nonlinear or threshold-driven ecological transitions. While the 10 km grid resolution balances spatial coverage and analytical tractability, it may obscure fine-scale habitat heterogeneity relevant for local management. Finally, our focus on southern vascular plant assemblages may limit direct generalization to other taxonomic groups or biogeographic contexts^5,67^.

Future research could address these limitations by integrating functional trait, phylogenetic, or ecosystem service layers, enhancing ecological realism and practical relevance^76–78^. For example, linking compositional shifts to ecosystem functions such as carbon sequestration or hydrological regulation could guide restoration decisions beyond biodiversity goals^79,80^. Incorporating social–ecological overlays, such as land ownership, policy zones, and governance regimes, can further bridge the gap between ecological predictions and on-ground implementation^81^. Methodologically, coupling this framework with process-based dynamic vegetation models or species interaction networks could capture additional complexity, including feedbacks, facilitation, or competitive displacement^82,83^. Comparative application across biogeographic regions or climatic zones would also help test the generality of the cluster typology observed here. For instance, unstable clusters like Cluster 8 (characterized by strong directional misalignment and high scenario divergence) could be prioritized for targeted ecological surveillance using drone-based hyperspectral imagery^84^, vegetation plot resurveys^85^, or eDNA sampling to validate compositional forecasts^86^ and detect early signals of novel ecosystem assembly^87^. Finally, empirical monitoring, especially in unstable clusters, can validate model predictions, offering evidence-based inputs for early-warning systems.

## Conclusion

In conclusion, we present a spatially explicit SDM-based framework that integrates hotspot mapping and compositional trajectory analysis to forecast climate-driven community reassembly of southern plant assemblages in South Korea. Beyond identifying where change is projected to occur, our approach also characterizes whether compositional shifts are directionally coherent (or incongruent) with underlying climatic gradients, enabling differentiation of persistence cores, transition zones, and high-uncertainty reorganization regions. These findings suggest that conservation and restoration planning under climate change may benefit from complementing static approaches with greater attention to dynamic community trajectories. In practice, this can support climate-refugia monitoring, connectivity-oriented management in transitional areas, and adaptive strategies in regions prone to transformational change. By providing an interpretable, spatially explicit decision-support layer aligned with GBF reporting and national planning needs, this framework can help inform climate-resilient biodiversity management under ongoing environmental change.

## Supplementary Information

Below is the link to the electronic supplementary material.

Supplementary Material 1

Supplementary Material 2

## Data Availability

The analysis code and summary datasets are provided as Supplementary Information. Further details can be obtained from the corresponding author upon reasonable request.
