# Supplementary material for: Projected compositional reorganization of Southern plant assemblages in South Korea under climate scenarios using species distribution models
Source: Sci Rep. 2026 Mar 14;16:13760. doi: 10.1038/s41598-026-44558-6 (PMC13129097; doi:10.1038/s41598-026-44558-6)
Supplement: Supplementary file 1 — Supplementary Material 1 [file 41598_2026_44558_MOESM1_ESM.pdf]

```
# ============================================================
# Full Analytical Workflow for:
# "Ecological Zone Dynamics of Southern Plant Assemblages in South Korea: Mapping Richness and Compositional Change under Climate Scenarios"
# ============================================================
# Authors: Kim, S-J., Lim, C.H.
# Date: 22 September 2025
# This script reproduces the complete analytical pipeline used in the current study, including:
#     - Richness-weighted kernel density estimation (KDE) for identifying core response zones
#     - Spatial clustering of species richness and composition patterns from SDM outputs
#     - Ordination-based trajectory analysis of community-level shifts across climate scenarios
#     - Climatic niche shift visualization in Whittaker bioclimatic space

# Note:
# Species distribution modeling (SDM) outputs used as input for the above analyses
# were generated following the methods described in the previous publication:

# Cho, Y.C., Seol, J., & Lim, C.H. (2024).
# "Climate-induced distribution dynamics and niche adaptation of South Korean endemic plants across the Korean Peninsula."
# *Scientific Reports*, 14, Article number: 22253. https://doi.org/10.1038/s41598-024-22253-6

# For model structure, parameter settings, and preprocessing steps,
# refer to Supplementary Data 1 of the above article.

# Climate scenarios:
#     - 126 = SSP1–2.6
#     - 370 = SSP3–7.0
#     - 585 = SSP5–8.5

# Time periods:
#     - Current (1980–2010)
#     - 1140 (2010–2040)
#     - 4170 (2040–2070)
#     - 7100 (2070–2100)
```

```
# Spatial resolution:
#     - 10 km × 10 km grid across South Korea

# Computational environment:
#     - R version 4.5.1
#     - Key packages: terra, sf, ks, vegan, ecotraj, dplyr, tidyr, purrr, ggplot2
# ==========================================================

  ==========================================================
# 1. Spatial Clustering of Environmentally Filtered Distribution Points
# ==========================================================

# Required packages
library(terra)
library(sf)
library(dplyr)
library(tidyverse)
library(mgcv)
library(readr)
library(scales)
library(cluster)

# Load environmental raster bundle and boundary
bundle_ras <- rast("Bundle.tif")
boundary     <- vect("Boundary.shp")

# Project and crop environmental rasters
bundle_mask <- mask(crop(bundle_ras, boundary), boundary)
bundle_proj <- project(bundle_mask, "EPSG:5186", method = "near")

# Aggregate to 10 km resolution
target_resolution <- 10000
fac <- round(target_resolution / res(bundle_proj)[1])
```

```
r_agg <- aggregate(bundle_proj, fact = fac, fun = mean, na.rm = TRUE)

# Create cell dataframe
cell_df <- as.data.frame(xyFromCell(r_agg, 1:ncell(r_agg)))
cell_df$cell_id <- 1:ncell(r_agg)
cell_df <- cell_df %>% rename(x = x, y = y)

# Load and project elevation raster
elev_r <- rast("F1_elevation.tif") %>% project("EPSG:5186", method = "bilinear")
boundary_proj <- project(boundary, crs(elev_r))
elev_crop <- crop(elev_r, boundary_proj) %>% mask(boundary_proj) %>% project("EPSG:5186", method =
"bilinear")
elev_10km <- resample(elev_crop, r_agg, method = "bilinear")

# Load and project sea distance raster
sea_r <- rast("SeaDist.tif") %>% project("EPSG:5186", method = "bilinear")
boundary_proj <- project(boundary, crs(sea_r))
sea_crop <- crop(sea_r, boundary_proj) %>% mask(boundary_proj) %>% project("EPSG:5186", method =
"bilinear")
sea_10km <- resample(sea_crop, r_agg, method = "bilinear")

# Load distribution points
sp <- readRDS("group_results_rds/coord_Southern_Current_Current.rds")
pts <- vect(sp, geom = c("x", "y"), crs = crs(elev_r))

# Extract mean elevation and sea distance per point
sp$elevation <- terra::extract(elev_r, pts, buffer = 5000, fun = mean, na.rm = TRUE)[,2]
sp$sea_dist   <- terra::extract(sea_r, pts, buffer = 5000, fun = mean, na.rm = TRUE)[,2]
sp$latitude   <- sp$y

# Elevation gap-filling using GAM
sp_nonmiss <- sp %>% filter(!is.na(elevation), !is.na(sea_dist))
fit_gam <- gam(elevation ~ s(sea_dist), data = sp_nonmiss)
sp$elevation_gamfill <- ifelse(
  is.na(sp$elevation) & !is.na(sp$sea_dist),
```

```
  predict(fit_gam, newdata = sp),
  sp$elevation
)

# Assemble clustering input
clu_df <- sp %>%
  dplyr::mutate(longitude = x) %>%
  dplyr::select(cell_id, x, y, longitude, elevation_gamfill, sea_dist, latitude) %>%
  filter(!is.na(elevation_gamfill), !is.na(sea_dist), !is.na(latitude), !is.na(longitude))

# Reproject for longitude/latitude extraction
clu_sf <- st_as_sf(clu_df, coords = c("x", "y"), crs = 5186)
clu_sf_wgs <- st_transform(clu_sf, 4326)
coords_deg <- st_coordinates(clu_sf_wgs)
clu_df$longitude <- coords_deg[, 1]
clu_df$latitude  <- coords_deg[, 2]

# K-means clustering
vars <- clu_df %>% dplyr::select(elevation_gamfill, sea_dist, latitude, longitude)
vars_scaled <- scale(vars)
sil_scores <- sapply(2:12, function(k) {
  km_res <- kmeans(vars_scaled, centers = k, nstart = 10)
  ss <- silhouette(km_res$cluster, dist(vars_scaled))
  mean(ss[, 3])
})
n_cluster <- which.max(sil_scores)
clu_df$cluster <- as.factor(kmeans(vars_scaled, centers = n_cluster)$cluster)

# Cluster summary with z-scores
clu_df <- clu_df %>% mutate(sea_dist_km = sea_dist / 1000)
global_stats <- clu_df %>%
  summarise(
    lat_mu = mean(latitude), lat_sd = sd(latitude),
    lon_mu = mean(longitude), lon_sd = sd(longitude),
```

```
    elev_mu = mean(elevation_gamfill), elev_sd = sd(elevation_gamfill),
    sea_mu = mean(sea_dist_km), sea_sd = sd(sea_dist_km)
  )

summary_tbl <- clu_df %>%
  group_by(cluster) %>%
  summarise(
    lat_mean = mean(latitude), lat_sd = sd(latitude),
    lon_mean = mean(longitude), lon_sd = sd(longitude),
    elev_mean = mean(elevation_gamfill), elev_sd = sd(elevation_gamfill),
    sea_mean = mean(sea_dist_km), sea_sd = sd(sea_dist_km),
    n_cells = n()
  ) %>%
  mutate(
    lat_fmt = paste0(round(lat_mean, 2), " (", round(lat_sd, 2), ")"),
    lon_fmt = paste0(round(lon_mean, 2), " (", round(lon_sd, 2), ")"),
    elev_fmt = paste0(round(elev_mean, 1), " (", round(elev_sd, 1), ")"),
    sea_fmt = paste0(round(sea_mean, 1), " (", round(sea_sd, 1), ")"),
    z_lat = round((lat_mean - global_stats$lat_mu) / global_stats$lat_sd, 2),
    z_lon = round((lon_mean - global_stats$lon_mu) / global_stats$lon_sd, 2),
    z_elev = round((elev_mean - global_stats$elev_mu) / global_stats$elev_sd, 2),
    z_sea = round((sea_mean - global_stats$sea_mu) / global_stats$sea_sd, 2)
  ) %>%
  select(
    cluster, lat_fmt, z_lat, lon_fmt, z_lon,
    elev_fmt, z_elev, sea_fmt, z_sea, n_cells
  ) %>%
  arrange(cluster)

# =====================================================
# 2. Compositional Trajectory Analysis with Repeated Sampling
# =====================================================

# Required packages
```

```
library(tidyverse)
library(vegan)
library(ecotraj)
library(broom)
library(glue)
library(permute)
library(jsonlite)

# Parameters
output_dir <- "Southern"
n_rep <- 30
cell_per_cluster <- 20
species_cols <- 9:103
period_levels <- c("Current", "1140", "4170", "7100")

# 1. Load and prepare data
load_and_clean_data <- function() {
  rds_files <- list.files("group_results_rds", pattern = "^sp_Southern_.*\\.rds$", full.names = TRUE)
  sp_all <- map_dfr(rds_files, readRDS)
  clu_df <- read_csv("clu_df.csv")
  sp_all <- left_join(sp_all, clu_df %>% select(cell_id, cluster), by = "cell_id") %>%
    filter(!(scenario == "Current" & period == "Current")) %>%
    mutate(
      period = factor(period, levels = period_levels, ordered = TRUE),
      scenario = as.factor(scenario),
      cluster = as.factor(cluster),
      traj_group = paste0("cl", cluster, "-", scenario)
    )
  sp_species <- sp_all[, species_cols]
  sp_all_clean <- sp_all[rowSums(sp_species) > 0, ]
  return(sp_all_clean)
}

# 2. Repeated trajectory-based analysis
```

```
run_repeated_analysis <- function(sp_all_clean, n_rep, cell_per_cluster, species_cols, period_levels,
output_dir) {
  all_results <- vector("list", n_rep)
  for (i in seq_len(n_rep)) {
    set.seed(i * 1000)

    valid_cells <- sp_all_clean %>%
      group_by(cluster, cell_id) %>%
      summarise(n_combo = n_distinct(paste(scenario, period)), .groups = "drop") %>%
      filter(n_combo == 12)

    sampled_cells <- valid_cells %>%
      group_by(cluster) %>%
      sample_n(cell_per_cluster, replace = FALSE) %>%
      pull(cell_id)

    analysis_data <- sp_all_clean %>%
      filter(cell_id %in% sampled_cells) %>%
      mutate(
        period_num = as.integer(factor(period, levels = period_levels)),
        traj_group = paste(cell_id, scenario, sep = "_")
      )

    sp_mat <- analysis_data[, species_cols]
    bc_dist <- vegdist(sp_mat, method = "bray")

    traj <- defineTrajectories(
      d = bc_dist,
      sites = analysis_data$traj_group,
      surveys = analysis_data$period_num,
      times = analysis_data$period_num
    )

    len_df <- trajectoryLengths(traj)
```

```
  dir_df <- trajectoryDirectionality(traj)
  var_df <- trajectoryInternalVariation(traj)
  seg_dist <- segmentDistances(traj)
  decomp_df <- variationDecomposition(traj)

  pcoa_res <- cmdscale(bc_dist, k = 2, eig = TRUE)
  pcoa_df <- as.data.frame(pcoa_res$points) %>%
    setNames(c("PCoA1", "PCoA2")) %>%
    bind_cols(analysis_data %>% select(cell_id, cluster, scenario, period))

  decomp_list <- list()
  for (cl in unique(analysis_data$cluster)) {
    data_cl <- analysis_data %>% filter(cluster == cl)
    sp_mat_cl <- data_cl[, species_cols]
    bc_dist_cl <- vegdist(sp_mat_cl, method = "bray")
    traj_cl <- defineTrajectories(
      d = bc_dist_cl,
      sites = data_cl$cell_id,
      surveys = data_cl$period_num,
      times = data_cl$period_num
    )
    decomp_list[[as.character(cl)]] <- variationDecomposition(traj_cl)
  }

  all_results[[i]] <- list(
    len_df = len_df,
    dir_df = dir_df,
    var_df = var_df,
    seg_dist = seg_dist,
    decomp_df = decomp_df,
    pcoa_df = pcoa_df,
    decomp_list = decomp_list
  )
}
```

```
  return(all_results)
}

# 3. Summary wrapper
summarize_results <- function(all_results, sp_all_clean) {
  cluster_lookup <- sp_all_clean %>%
    select(cell_id, cluster) %>%
    mutate(cell_id = as.character(cell_id)) %>%
    distinct()

  unpack <- function(df_list, name, col) {
    map2_dfr(df_list, seq_along(df_list), function(res, i) {
      df <- res[[name]]
      df <- as.data.frame(df)
      df <- rownames_to_column(df, var = "cell_id") %>%
        separate(cell_id, into = c("cell_id_num", "scenario"), sep = "_", remove = FALSE) %>%
        mutate(
          replicate = i,
          scenario = as.character(scenario),
          cell_id_num = as.character(cell_id_num)
        ) %>%
        left_join(cluster_lookup, by = c("cell_id_num" = "cell_id"))
      colnames(df)[colnames(df) == col] <- "value"
      df
    })
  }

  len_summary <- unpack(all_results, "len_df", "Path")
  dir_summary <- unpack(all_results, "dir_df", "directionality_vec")
  var_summary <- unpack(all_results, "var_df", "internal_variance")

  decomp_long <- map2_dfr(all_results, seq_along(all_results), function(res, i) {
    map_dfr(res$decomp_list, ~ mutate(as.data.frame(.x), effect = rownames(.x)), .id = "cluster") %>%
      mutate(replicate = i)
```

```
  }) %>%
    group_by(cluster, replicate) %>%
    mutate(prop = ss / sum(ss)) %>%
    ungroup()

  pcoa_all <- bind_rows(lapply(all_results, `[[`, "pcoa_df"), .id = "replicate")

  return(list(
    len_summary = len_summary,
    dir_summary = dir_summary,
    var_summary = var_summary,
    decomp_all_df = decomp_long,
    pcoa_all = pcoa_all
  ))
}

# 4. Run full pipeline
sp_all_clean <- load_and_clean_data()
all_results <- run_repeated_analysis(
  sp_all_clean = sp_all_clean,
  n_rep = n_rep,
  cell_per_cluster = cell_per_cluster,
  species_cols = species_cols,
  period_levels = period_levels,
  output_dir = output_dir
)
result_summary <- summarize_results(all_results, sp_all_clean)

# ============================================================
# 3. Alignment Analysis between Community Trajectories and Environmental Gradients
# ============================================================

# Required packages
library(dplyr)
```

```r
library(readr)
library(purrr)
library(vegan)
library(tidyr)

# 1. Load PCoA coordinates and metadata

output_dir <- "Southern"

pcoa_df <- read_csv(file.path(output_dir, "pcoa_all.csv"), show_col_types = FALSE) %>%
  mutate(
    replicate = if (!"replicate" %in% names(.)) 1L else as.integer(replicate),
    cell_id = as.character(cell_id),
    scenario = as.character(scenario),
    period = as.character(period)
  )

# 2. Load and merge environmental variables

env_files <- list.files("group_results_rds", pattern = "^env_Southern_.*\\.rds$", full.names = TRUE)

env_df <- map_dfr(env_files, ~{
  fname <- .x
  parts <- strsplit(basename(fname), "_")[[1]]
  scenario <- parts[3]
  period <- gsub("\\.rds$", "", parts[4])

  readRDS(fname) %>%
    mutate(
      scenario = scenario,
      period = period,
      cell_id = as.character(cell_id)
    )
}) %>%
```

```
  dplyr::select(cell_id, scenario, period, latitude, longitude, elevation, sea_dist) %>%
  filter(complete.cases(.))

# 3. Join coordinates with environmental data

pcoa_env <- left_join(pcoa_df, env_df, by = c("cell_id", "scenario", "period"))

# 4. Compute cosine(Δθ) between trajectory vector and envfit vector

calculate_cos_delta_theta <- function(traj, env_data) {
  env_vec <- envfit(traj$coord[, 1:2], env_data, permutations = 0)$vectors$arrows
  if (nrow(env_vec) == 0) return(rep(NA, nrow(traj$coord)))

  deltas <- traj$coord %>%
    group_by(traj_id) %>%
    summarise(
      dx = diff(PCoA1),
      dy = diff(PCoA2),
      .groups = "drop"
    )

  cos_vals <- sapply(1:nrow(env_vec), function(i) {
    v_env <- env_vec[i, ]
    v_traj <- cbind(deltas$dx, deltas$dy)
    dot_prod <- rowSums(v_env * v_traj)
    norm_env <- sqrt(sum(v_env^2))
    norm_traj <- sqrt(rowSums(v_traj^2))
    dot_prod / (norm_env * norm_traj)
  })

  cos_vals
}

# 5. Apply analysis across replicates
```

```
cos_theta_results <- pcoa_env %>%
  group_by(traj_group, replicate) %>%
  nest() %>%
  mutate(
    traj_id = paste(traj_group, replicate, sep = "_"),
    coords = map(data, ~ .x %>% arrange(period) %>% dplyr::select(PCoA1, PCoA2)),
    envs   = map(data, ~ .x %>% summarise(across(c(latitude, longitude, elevation, sea_dist), mean,
na.rm = TRUE))),
    cos_results = map2(coords, envs, calculate_cos_delta_theta)
  ) %>%
  unnest(cols = c(cos_results)) %>%
  dplyr::select(traj_group, replicate, cos_results)

# ==========================================================
# 4. Permutational Multivariate Analysis (PERMANOVA & PERMDISP) of Community Shifts
# ==========================================================

# Required packages
library(tidyverse)
library(vegan)
library(fs)
library(glue)
library(readr)
library(tibble)
library(purrr)

# Set directories and global parameters
output_dir <- "Southern_S1only"
dir_create(output_dir)
cache_dir  <- file.path(output_dir, "cache_sampling")
dir_create(cache_dir)

n_rep <- 30
```

```
cell_per_cluster <- 20
species_cols <- 9:103
period_levels <- c("Current", "1140", "4170", "7100")

# Analysis mode: "fast" for exploratory runs, "final" for full tables
RUN_MODE <- "fast"

if (RUN_MODE == "fast") {
  perm_global         <- 4999L
  perm_bycl           <- 1999L
  perm_permdisp       <- 999L
  by_mode             <- "margin"
  run_bycl_permanova        <- FALSE
  run_bycluster_permdisp <- FALSE
  include_interaction      <- FALSE
  include_scenario          <- FALSE
} else {
  perm_global         <- 4999L
  perm_bycl           <- 1999L
  perm_permdisp       <- 1999L
  by_mode             <- "margin"
  run_bycl_permanova        <- TRUE
  run_bycluster_permdisp <- TRUE
  include_interaction      <- TRUE
  include_scenario          <- FALSE
}

# Load cleaned data with species and metadata
sp_all_clean <- load_and_clean_data()

# Identify fully synchronized cell-period-scenario combinations
valid_cells <- sp_all_clean %>%
  group_by(cluster, cell_id) %>%
  summarise(n_combo = n_distinct(paste(scenario, period)), .groups = "drop") %>%
```

```
  filter(n_combo == 12)

# Caching sampled cell IDs for reproducibility
sampling_file <- function(rep_id) file.path(cache_dir, sprintf("cells_rep%02d.rds", rep_id))

get_cells_for_rep <- function(rep_id, cell_per_cluster) {
  f <- sampling_file(rep_id)
  if (file_exists(f)) return(readRDS(f))
  set.seed(rep_id * 1000)
  cells <- valid_cells %>%
    group_by(cluster) %>%
    sample_n(cell_per_cluster, replace = FALSE) %>%
    ungroup() %>%
    pull(cell_id)
  saveRDS(cells, f)
  cells
}

# Tidy wrapper for adonis2 result
tidy_adonis2_df <- function(fit, replicate = NA_integer_, drop_rows = c("Total", "Residual")) {
  df <- as.data.frame(fit)
  df$term <- rownames(df)
  pick <- function(cands) {
    ix <- match(cands, tolower(names(df)))
    if (all(is.na(ix))) stop("Missing column in adonis2 result.")
    names(df)[na.omit(ix)[1]]
  }
  tibble(
    Effect     = df$term,
    R2         = as.numeric(df[[pick(c("r2", "r.squared"))]]),
    F          = suppressWarnings(as.numeric(df[[pick(c("f", "f value"))]])),
    p_perm     = suppressWarnings(as.numeric(df[[pick(c("pr(>f)", "p.value"))]])),
    replicate = replicate
  ) %>% filter(!Effect %in% drop_rows)
```

```
}

# PERMANOVA computation function
compute_S1_tables <- function(
  sp_mat, bc_dist, analysis_data, rep_id,
  period_levels = c("Current", "1140", "4170", "7100"),
  perm_global = 9999L, perm_bycl = 1999L, perm_permdisp = 1999L,
  by_mode = "margin",
  run_bycl_permanova = TRUE,
  run_bycluster_permdisp = TRUE,
  include_interaction = FALSE,
  include_scenario = FALSE
) {
  dat <- analysis_data %>%
    mutate(period = factor(period, levels = period_levels, ordered = TRUE),
           cluster = factor(cluster),
           cell_id = as.factor(cell_id))

  cl_levels <- levels(dat$cluster)
  form_global <- if (include_scenario) {
    if (include_interaction) bc_dist ~ period + cluster + scenario + period:cluster
    else bc_dist ~ period + cluster + scenario
  } else {
    if (include_interaction) bc_dist ~ period + cluster + period:cluster
    else bc_dist ~ period + cluster
  }

  # (a) Global PERMANOVA
  fit_global <- adonis2(form_global, data = dat, permutations = perm_global,
                        strata = dat$cell_id, by = by_mode)
  tab_global <- tidy_adonis2_df(fit_global, replicate = rep_id)

  # (b) Cluster-specific PERMANOVA
  tabs_bycl <- if (run_bycl_permanova && perm_bycl > 0L) {
```

```
  map_dfr(cl_levels, function(clj) {
    ix <- which(dat$cluster == clj)
    if (length(ix) < 2 || length(unique(dat$period[ix])) < 2) return(NULL)
    dij <- as.dist(as.matrix(bc_dist)[ix, ix])
    fit_j <- adonis2(dij ~ period, data = dat[ix, ],
                     permutations = perm_bycl, strata = dat$cell_id[ix], by = by_mode)
    tidy_adonis2_df(fit_j, replicate = rep_id) %>% mutate(Cluster = clj)
  })
} else tibble()

# (c) PERMDISP (overall)
if (perm_permdisp > 0L) {
  bd <- betadisper(bc_dist, group = dat$period)
  pt <- permutest(bd, permutations = perm_permdisp)
  tab_disp <- tibble(
    Test      = c("PERMDISP_ANOVA", "PERMDISP_Perm"),
    F         = c(anova(bd)[1, "F"], pt$tab[1, "F"]),
    p_perm    = c(anova(bd)[1, "Pr(>F)"], pt$tab[1, "Pr(>F)"]),
    replicate = rep_id
  )
} else {
  tab_disp <- tibble(Test = c("PERMDISP_ANOVA", "PERMDISP_Perm"),
                     F = NA_real_, p_perm = NA_real_, replicate = rep_id)
}

# (d) PERMDISP by cluster
tabs_disp_bycl <- if (run_bycluster_permdisp && perm_permdisp > 0L) {
  map_dfr(cl_levels, function(clj) {
    ix <- which(dat$cluster == clj)
    grp <- droplevels(dat$period[ix])
    if (length(ix) < 3 || length(unique(grp)) < 2) return(NULL)
    dij <- as.dist(as.matrix(bc_dist)[ix, ix])
    bdj <- betadisper(dij, group = grp)
    pj  <- permutest(bdj, permutations = perm_permdisp)
```

```
      tibble(
        Cluster    = clj,
        Test       = c("PERMDISP_ANOVA", "PERMDISP_Perm"),
        F          = c(anova(bdj)[1, "F"], pj$tab[1, "F"]),
        p_perm     = c(anova(bdj)[1, "Pr(>F)"], pj$tab[1, "Pr(>F)"]),
        replicate = rep_id
      )
    })
  } else tibble()

  return(list(
    global = tab_global,
    by_cluster = tabs_bycl,
    permdisp = tab_disp,
    permdisp_bycl = tabs_disp_bycl
  ))
}

# Main loop: replicate-wise community shift analysis
for (i in seq_len(n_rep)) {
  sampled_cells <- get_cells_for_rep(i, cell_per_cluster)

  analysis_data_raw <- sp_all_clean %>%
    filter(cell_id %in% sampled_cells) %>%
    mutate(period = factor(period, levels = period_levels, ordered = TRUE))

  sp_cols <- colnames(analysis_data_raw)[species_cols]
  analysis_data_avg <- analysis_data_raw %>%
    group_by(cell_id, cluster, period) %>%
    summarise(across(all_of(sp_cols), mean, na.rm = TRUE), .groups = "drop")

  sp_mat <- as.matrix(analysis_data_avg[, sp_cols])
  sp_mat <- sp_mat[, colSums(sp_mat, na.rm = TRUE) > 0, drop = FALSE]
  bc_dist <- vegdist(sp_mat, method = "bray")
```

```
  form_quick <- bc_dist ~ period + cluster + period:cluster
  fit_quick <- adonis2(form_quick, data = analysis_data_avg,
                       permutations = 499, strata = analysis_data_avg$cell_id,
                       by = "terms")
  tab_quick <- as.data.frame(fit_quick)
  p_gate <- if ("period:cluster" %in% rownames(tab_quick)) tab_quick["period:cluster", "Pr(>F)"] else
NA_real_

  run_bycl_now <- is.finite(p_gate) && p_gate < 0.10
  perm_global_use <- if (is.finite(p_gate) && p_gate > 0.02 && p_gate <= 0.10) {
    max(perm_global, 4999L)
  } else perm_global

  invisible(compute_S1_tables(
    sp_mat, bc_dist, analysis_data = analysis_data_avg, rep_id = i,
    perm_global = perm_global_use,
    perm_bycl = if (run_bycl_now) max(perm_bycl, 1999L) else 0L,
    perm_permdisp = perm_permdisp,
    by_mode = by_mode,
    run_bycluster_permdisp = run_bycluster_permdisp,
    run_bycl_permanova = run_bycl_now,
    include_scenario = include_scenario,
    include_interaction = include_interaction
  ))
}

# ============================================================
# 5. Kernel Density Estimation (KDE) for Richness-Based Core Area Detection
# ============================================================

# Require libraries
library(tidyverse)    # Includes dplyr, purrr, readr, ggplot2, etc.
library(sf)
```

```
library(ks)              # For Hpi, kde, contourLevels
options(sf_use_s2 = FALSE)

period_levels <- c("Current","1140","4170","7100")
species_cols   <- 9:103
crs_target     <- 5186
out_dir        <- "kde50_outputs"
dir.create(out_dir, showWarnings = FALSE)

# 1. Load and clean data
load_and_clean_data <- function() {
  rds_files <- list.files("group_results_rds", pattern="^sp_Southern_.*\\.rds$", full.names=TRUE)
  stopifnot(length(rds_files) > 0)

  sp_all <- purrr::map_dfr(rds_files, readRDS)
  clu_df <- readr::read_csv("clu_df.csv", show_col_types = FALSE) %>% select(cell_id, cluster)

  sp_all <- sp_all %>%
    left_join(clu_df, by="cell_id") %>%
    filter(!(scenario == "Current" & period == "Current")) %>%
    mutate(
      period     = factor(period, levels = period_levels, ordered = TRUE),
      scenario = factor(scenario),
      cluster   = factor(cluster)
    )

  sp_species <- sp_all[, species_cols]
  keep_idx     <- rowSums(sp_species) > 0
  sp_all_clean <- sp_all[keep_idx, ]
  sp_all_clean$richness <- rowSums(sp_all_clean[, species_cols] > 0, na.rm = TRUE)

  sp_all_clean
}
sp_all_clean <- load_and_clean_data()
```

2. KDE function

```
get_kde_poly <- function(dat,
                         xcol = "x", ycol = "y",
                         wcol = "richness",
                         prob = 0.5,
                         crs = crs_target,
                         min_pts = 5) {
  dat <- dat %>% filter(!is.na(.data[[xcol]]), !is.na(.data[[ycol]]))
  if (nrow(dat) < min_pts) return(NULL)

  xy <- as.matrix(dat[, c(xcol, ycol)])
  w_raw <- as.numeric(dat[[wcol]])
  if (all(is.na(w_raw)) || all(w_raw <= 0)) w_raw <- rep(1, nrow(dat))
  w <- pmax(1, round(w_raw))

  # Bandwidth estimation using replicated weights
  H <- ks::Hpi(x = xy[rep(seq_len(nrow(xy)), w), , drop = FALSE], binned = TRUE)
  kd <- ks::kde(x = xy, H = H, w = w, compute.cont = TRUE)

  lvl <- ks::contourLevels(kd, prob = prob, approx = TRUE)
  cl   <- with(kd, contourLines(eval.points[[1]], eval.points[[2]], estimate, levels = lvl))
  if (length(cl) == 0) return(NULL)

  polys <- lapply(cl, \(c) st_polygon(list(cbind(c$x, c$y))))
  st_sf(level = sprintf("%.2f", prob),
        geometry = st_sfc(polys, crs = crs)) |> st_make_valid()
}
```

3. KDE estimation by scenario × period

```
scenarios <- levels(sp_all_clean$scenario)
periods    <- period_levels
```

```
poly_list <- list()
for (sc in scenarios) for (pe in periods) {
  dat <- sp_all_clean %>% filter(scenario == sc, period == pe)
  kde50 <- get_kde_poly(dat, prob = 0.5)
  nm <- paste0(sc, "_", pe)
  if (is.null(kde50)) next
  kde50$scenario <- as.character(sc)
  kde50$period    <- as.character(pe)
  poly_list[[nm]] <- kde50
}
all_kde50 <- dplyr::bind_rows(poly_list) |> st_make_valid() |> st_cast("MULTIPOLYGON", warn = FALSE)


# 4. Clip to land boundary and summarize area

bnd_land <- st_read("Boundary.shp", quiet = TRUE) |>
  st_transform(crs_target) |>
  st_make_valid() |>
  st_union()

# First bounding box clip, then exact intersection
kde50_v <- st_intersection(all_kde50, st_as_sfc(st_bbox(bnd_land)))
all_kde50_clip <- st_intersection(kde50_v, bnd_land) |>
  subset(!st_is_empty(geometry))

# Area summary (km²)
area_tbl <- all_kde50_clip %>%
  mutate(area_km2 = as.numeric(st_area(geometry)) / 1e6) %>%
  st_drop_geometry() %>%
  group_by(scenario, period) %>%
  summarise(n_poly = n(),
            total_area_km2   = sum(area_km2, na.rm = TRUE),
            median_area_km2 = median(area_km2, na.rm = TRUE),
```

```r
                    .groups = "drop")

# ======================================================
# 6. Extract Metrics from KDE50 Polygons (Area, Centroid, Elevation, Distance)
# ======================================================

# Load Required Packages
library(sf); library(dplyr); library(tidyr); library(ggplot2)
library(terra); library(units); library(scales)
has_exact <- requireNamespace("exactextractr", quietly = TRUE)

# Constants
period_levels <- c("Current","1140","4170","7100")

# 1. Preprocess Spatial Inputs (Union of KDE50 Polygons, Raster Load)

# KDE50 union by scenario × period (EPSG:5186)
kde_u_5186 <- all_kde50_clip |>
  st_make_valid() |>
  group_by(scenario, period) |>
  summarise(geometry = st_union(geometry), .groups = "drop") |>
  st_make_valid()

# Raster preparation
elev_5186 <- terra::project(terra::rast("f1_elevation.tif"), "EPSG:5186", method = "bilinear")
sea_5186   <- if (file.exists("SeaDist.tif")) terra::project(terra::rast("SeaDist.tif"), "EPSG:5186", method =
"bilinear") else NULL

# 2. Utility Functions

# Compute latitude quantiles (q10, q50, q90) for a polygon
lat_quants <- function(g, res_m = 3000) {
  g_5186 <- st_transform(g, 5186)
  r <- terra::rast(ext(terra::vect(g_5186)), resolution = res_m, crs = "EPSG:5186")
```

```
  m <- terra::mask(terra::rasterize(terra::vect(g_5186), r, field = 1), terra::vect(g_5186))
  idx <- which(!is.na(values(m)))
  if (length(idx) == 0) return(c(q10=NA, q50=NA, q90=NA))
  xy <- terra::xyFromCell(m, idx)
  xy_ll <- sf::st_coordinates(sf::st_transform(st_as_sf(data.frame(x=xy[,1], y=xy[,2]), coords = c("x","y"), crs = 5186), 4326))
  qs <- quantile(xy_ll[,2], probs = c(.10,.50,.90), na.rm = TRUE)
  c(q10 = unname(qs[1]), q50 = unname(qs[2]), q90 = unname(qs[3]))
}

# Compute mean, median, IQR for raster values within polygons
rast_summ <- function(r, polys_5186) {
  if (is.null(r)) return(list(mean=rep(NA,nrow(polys_5186)), median=rep(NA,nrow(polys_5186)), iqr=rep(NA,nrow(polys_5186))))
  if (has_exact) {
    mean_v <- exactextractr::exact_extract(r, polys_5186, "mean")
    med_v <- exactextractr::exact_extract(r, polys_5186, function(v, cf) stats::weighted.quantile(v, cf, probs=0.5, na.rm=TRUE))
    q25_v <- exactextractr::exact_extract(r, polys_5186, function(v, cf) stats::weighted.quantile(v, cf, probs=0.25, na.rm=TRUE))
    q75_v <- exactextractr::exact_extract(r, polys_5186, function(v, cf) stats::weighted.quantile(v, cf, probs=0.75, na.rm=TRUE))
    iqr_v <- q75_v - q25_v
  } else {
    mean_v <- terra::extract(r, terra::vect(polys_5186), fun = mean, na.rm = TRUE)[,2]
    get_vec <- function(i){
      v <- terra::extract(r, terra::vect(polys_5186[i,]), cells = TRUE)[,3]
      v <- v[!is.na(v)]
      if (length(v) == 0) return(c(med=NA, q25=NA, q75=NA))
      c(med=median(v), q25=quantile(v, .25), q75=quantile(v, .75))
    }
    qmat <- t(vapply(seq_len(nrow(polys_5186)), get_vec, numeric(3)))
    med_v <- qmat[,"med"]
    iqr_v <- qmat[,"q75"] - qmat[,"q25"]
  }
  list(mean=as.numeric(mean_v), median=as.numeric(med_v), iqr=as.numeric(iqr_v))
```

```
}

# 3. Metric Extraction: Area, Centroid, Elevation, Distance to Sea

# Area (km²)
area_km2 <- as.numeric(set_units(st_area(kde_u_5186), "km^2"))

# Centroids and latitude quantiles
kde_u_ll <- st_transform(kde_u_5186, 4326)
cent_xy <- st_coordinates(st_centroid(kde_u_ll))
lat_q <- t(vapply(st_geometry(kde_u_ll), lat_quants, numeric(3)))
colnames(lat_q) <- c("lat_q10", "lat_q50", "lat_q90")

# Raster summaries
elev_s <- rast_summ(elev_5186, kde_u_5186)
sea_s   <- rast_summ(sea_5186,   kde_u_5186)

# Combined metrics table
metrics_tbl <- kde_u_ll |>
  st_drop_geometry() |>
  mutate(
    period          = factor(period, levels = period_levels, ordered = TRUE),
    area_km2        = area_km2,
    centroid_lon    = cent_xy[,1],
    centroid_lat    = cent_xy[,2],
    lat_q10         = lat_q[,"lat_q10"],
    lat_q50         = lat_q[,"lat_q50"],
    lat_q90         = lat_q[,"lat_q90"],
    elev_mean_m     = elev_s$mean,
    elev_median_m = elev_s$median,
    elev_iqr_m      = elev_s$iqr,
    sea_mean_m      = sea_s$mean,
    sea_median_m    = sea_s$median,
    sea_iqr_m       = sea_s$iqr
```

```
  ) |>
  arrange(scenario, period)

# 4. Delta Calculation and Centroid Shift (Current vs 7100)

# Extract relevant columns for delta comparison
delta_tbl <- metrics_tbl |>
  select(scenario, period,
         centroid_lat, centroid_lon, lat_q90,
         area_km2, elev_median_m, elev_iqr_m, sea_median_m, sea_iqr_m) |>
  pivot_wider(names_from = period,
              values_from = c(centroid_lat, centroid_lon, lat_q90,
                              area_km2, elev_median_m, elev_iqr_m, sea_median_m, sea_iqr_m)) |>
  mutate(
    d_centroid_lat = centroid_lat_7100 - centroid_lat_Current,
    d_centroid_lon = centroid_lon_7100 - centroid_lon_Current,
    d_lat_q90      = lat_q90_7100      - lat_q90_Current,
    d_area_km2     = area_km2_7100     - area_km2_Current,
    d_elev_med_m   = elev_median_m_7100 - elev_median_m_Current,
    d_sea_med_m    = sea_median_m_7100  - sea_median_m_Current,
    d_elev_iqr_m   = elev_iqr_m_7100    - elev_iqr_m_Current,
    d_sea_iqr_m    = sea_iqr_m_7100     - sea_iqr_m_Current
  )

# Centroid shift (km)
cent_cur <- metrics_tbl |> filter(period=="Current") |> select(scenario, lonC=centroid_lon, latC=centroid_lat)
cent_fut <- metrics_tbl |> filter(period=="7100")    |> select(scenario, lonF=centroid_lon, latF=centroid_lat)

arrow_df <- left_join(cent_cur, cent_fut, by="scenario") |>
  rowwise() |>
  mutate(shift_km = {
    p1 <- st_sfc(st_point(c(lonC, latC)), crs=4326)
    p2 <- st_sfc(st_point(c(lonF, latF)), crs=4326)
    as.numeric(set_units(st_distance(p1, p2), "km"))
```

```r
  }) |>
  ungroup()

delta_tbl <- left_join(delta_tbl, arrow_df |> select(scenario, shift_km), by="scenario")

# ============================================================
# 7. Climate Space Shift and Biome Overlap Using Whittaker Diagram Framework
# ============================================================

# Required Packages
library(terra)
library(sf)
library(dplyr)
library(readr)
library(ggplot2)
library(ggrepel)
library(purrr)
library(plotbiomes)
library(ellipse)
library(tidyr)

# Constants and Inputs
period_levels <- c("Current","1140","4170","7100")
vars <- c("bio1","bio12")  # MAT, MAP
rescale_params <- list(
  bio1  = list(scale = 0.1, offset = -273.15),  # Convert to Celsius
  bio12 = list(scale = 0.1, offset = 0)          # Convert to mm
)

# 1. Load and Rescale CHELSA Bioclim Variables
load_stack <- function(base_dir, vars, tmpl, bnd_sf, rescale_params) {
  rast_list <- lapply(vars, function(v){
    fp <- file.path(base_dir, paste0("CHELSA_", v, ".grd"))
    r <- rast(fp) |> project(crs(tmpl), method="bilinear") |> resample(tmpl) |> mask(vect(bnd_sf))
```

```r
    p <- rescale_params[[v]]; r <- r * p$scale + p$offset; names(r) <- v; r
  })
  rast(rast_list)
}

# 2. Extract Cluster-wise Mean Values
extract_means <- function(stk, polys, period) {
  tb <- terra::extract(stk, terra::vect(polys), fun = mean, na.rm = TRUE) |> as_tibble()
  polys |> st_drop_geometry() |> mutate(row_id = row_number()) |>
    left_join(tb |> mutate(row_id = 1:n()), by="row_id") |>
    transmute(cluster = as.factor(cluster),
              period = factor(period, levels = c("Current", "7100")),
              MAT_C = bio1, MAP_cm = bio12 / 10)
}

# 3. Construct Bioclimatic Summary
pts_df <- bind_rows(
  extract_means(stk_cur, poly_t, "Current"),
  extract_means(stk_fut, poly_t, "7100")
) |> filter(is.finite(MAT_C), is.finite(MAP_cm))

clu_med <- pts_df |> group_by(cluster, period) |> summarise(
  MAT = median(MAT_C), MAP = median(MAP_cm), .groups = "drop"
)

arrow_df <- clu_med |> pivot_wider(names_from = period, values_from = c(MAT, MAP)) |>
  transmute(cluster, x0 = MAT_Current, y0 = MAP_Current,
                     x1 = MAT_7100,    y1 = MAP_7100)

# 4. Ellipse and Overlap Statistics (Current vs 7100)
S4_overall <- list(
  ellipse_stats(pts_df, level = 0.50, xvar = "MAT_C", yvar = "MAP_cm"),
  ellipse_stats(pts_df, level = 0.95, xvar = "MAT_C", yvar = "MAP_cm")
) |> bind_rows()
```

```
S4_byCluster <- split(pts_df, pts_df$cluster) |>
  map_dfr(~ bind_rows(
    ellipse_stats(.x, level = 0.50, xvar = "MAT_C", yvar = "MAP_cm"),
    ellipse_stats(.x, level = 0.95, xvar = "MAT_C", yvar = "MAP_cm")
  ), .id = "Cluster")

# 5. Compute Biome Proportions Within Climate Ellipses
x_lim <- c(7,21); y_lim <- c(75,300)
biome_sf <- extract_biome_polys(x_lim, y_lim)

ell_list <- list(
  cur50 = ell_poly_eig(filter(pts_df, period == "Current"), xvar="MAT_C", yvar="MAP_cm", level=0.50,
x_lim=x_lim, y_lim=y_lim),
  fut50 = ell_poly_eig(filter(pts_df, period == "7100"), xvar="MAT_C", yvar="MAP_cm", level=0.50,
x_lim=x_lim, y_lim=y_lim),
  cur95 = ell_poly_eig(filter(pts_df, period == "Current"), xvar="MAT_C", yvar="MAP_cm", level=0.95,
x_lim=x_lim, y_lim=y_lim),
  fut95 = ell_poly_eig(filter(pts_df, period == "7100"), xvar="MAT_C", yvar="MAP_cm", level=0.95,
x_lim=x_lim, y_lim=y_lim)
)

S4_biome_prop <- bind_rows(
  biome_share(ell_list$cur50, biome_sf) |> mutate(period="Current", level=50),
  biome_share(ell_list$fut50, biome_sf) |> mutate(period="7100", level=50),
  biome_share(ell_list$cur95, biome_sf) |> mutate(period="Current", level=95),
  biome_share(ell_list$fut95, biome_sf) |> mutate(period="7100", level=95)
) |> arrange(level, period, desc(prop))

# 6. Cluster-to-Cluster Overlap Analysis (95%)
E_cur <- make_ell_by_cluster(pts_df, "Current", 0.95, xvar="MAT_C", yvar="MAP_cm")
E_fut <- make_ell_by_cluster(pts_df, "7100", 0.95, xvar="MAT_C", yvar="MAP_cm")

overlap_tbl <- tidyr::expand_grid(cur = names(E_cur), fut = names(E_fut)) |>
  mutate(
```

```
  A_c = map_dbl(cur, ~ .area0(E_cur[[.x]])),
  A_f = map_dbl(fut, ~ .area0(E_fut[[.x]])),
  A_i = map2_dbl(cur, fut, ~ .area0(st_intersection(E_cur[[.x]], E_fut[[.y]]))),
  IoU    = if_else((A_c + A_f - A_i) > 0, A_i / (A_c + A_f - A_i), 0),
  prop_C = if_else(A_c > 0, A_i / A_c, 0),
  prop_F = if_else(A_f > 0, A_i / A_f, 0)
)
```
