# Supplementary material for: Projected compositional reorganization of Southern plant assemblages in South Korea under climate scenarios using species distribution models
Source: Sci Rep. 2026 Mar 14;16:13760. doi: 10.1038/s41598-026-44558-6 (PMC13129097; doi:10.1038/s41598-026-44558-6)
Supplement: Supplementary file 2 — Supplementary Material 2 [file 41598_2026_44558_MOESM2_ESM.pdf]

Supplementary Figure 1. Average silhouette width across candidate numbers of clusters (k = 2–15). The dashed line indicates the selected solution (k = 9). Silhouette values show a broad plateau rather than a sharp maximum, supporting the selection of k based on ecological interpretability rather than numerical optimization alone.

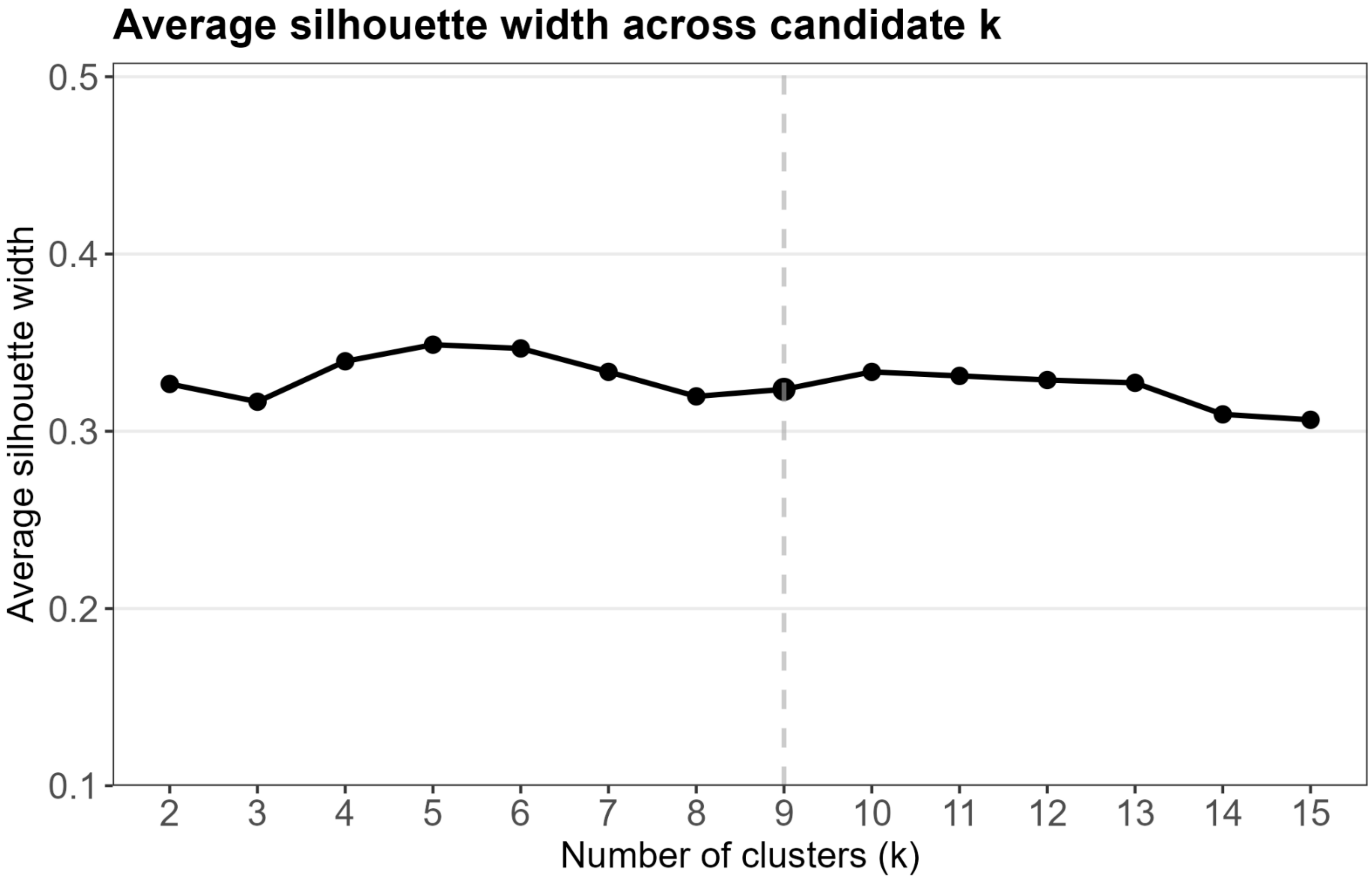

Supplementary Figure 2. Overlay of spatial clusters with national park boundaries in South Korea. Spatial distribution of the nine environmental clusters (Clusters 1–9) overlaid with the boundaries of national parks in South Korea. The map provides a park-scale spatial context to interpret cluster-based response types and to support practical conservation planning, monitoring design, and climate-adaptive management strategies. The map was generated using ArcGIS Pro version 3.2 (Esri Inc., https://www.esri.com/en-us/arcgis/products/arcgis-pro/overview). Administrative boundaries were obtained from GADM v4.1 (https://gadm.org).

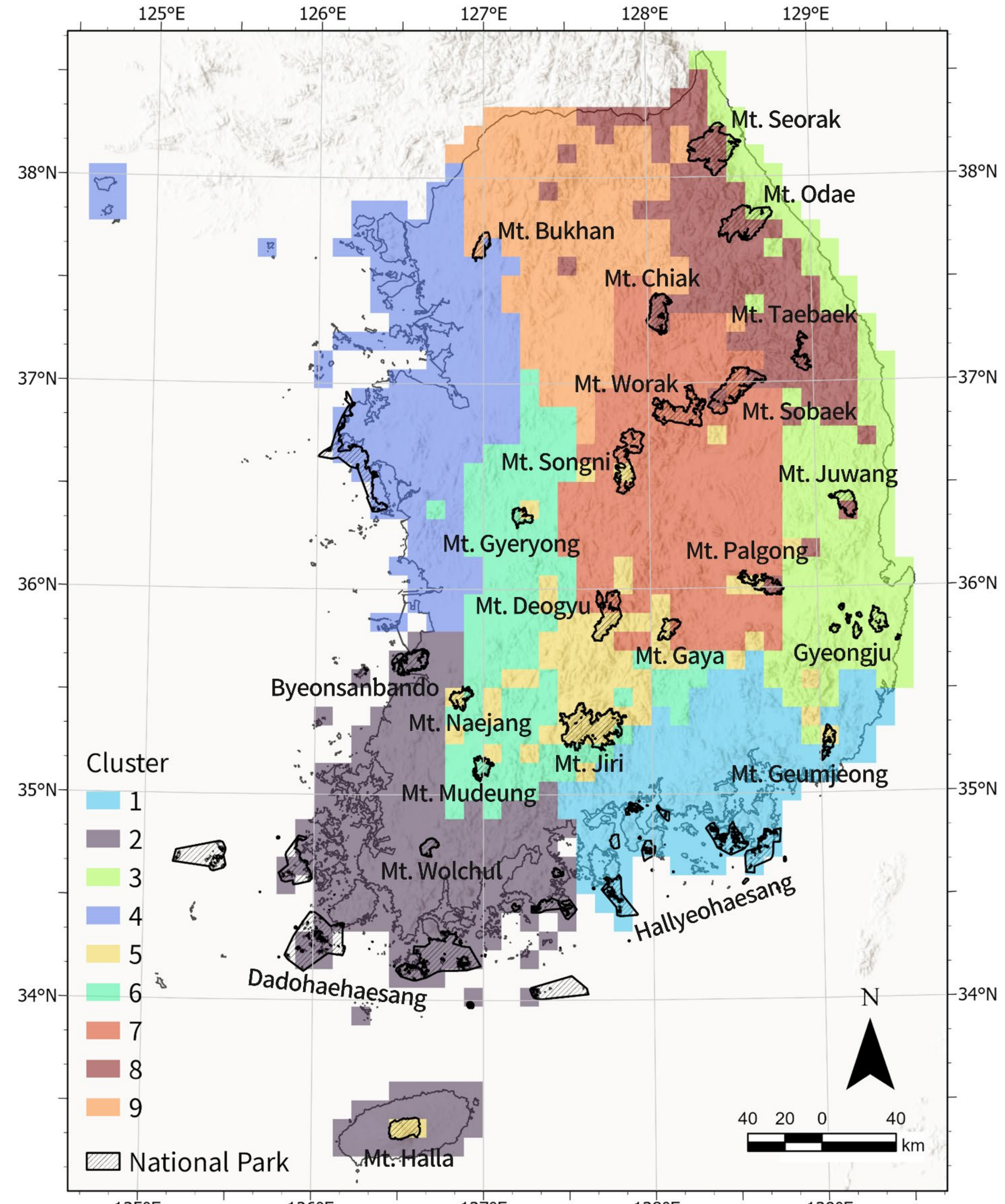

Supplementary Table 1. List of southern lineage plant species used in the analysis

* Note: Scientific names are italicized following binomial nomenclature.

Family	Genus	Species	Family	Genus	Species
Acanthaceae	*Strobilanthes*	Strobilanthes oligantha Miq.	*Lamiaceae*	*Salvia*	Salvia japonica Thunb.
	Actinidia	Actinidia rufa (Siebold & Zucc.) Planch. ex Miq.	*Lauraceae*	*Cinnamomum*	Cinnamomum chekiangense Nakai
Amaryllidaceae	*Lycoris*	Lycoris sanguinea Maxim. var. koreana (Nakai) T.Koyama		*Lindera*	Lindera sericea (Siebold & Zucc.) Blume
Anacardiaceae	*Toxicodendron*	Toxicodendron succedaneum (L.) Kuntze		*Actinodaphne*	Litsea coreana H.L?v.
		Toxicodendron sylvestre (Siebold & Zucc.) Kuntze		*Litsea*	Litsea japonica (Thunb.) Juss.
Apiaceae	*Angelica*	Angelica japonica A.Gray		*Machilus*	Machilus japonica Siebold & Zucc. ex Blume
	Cnidium	Cnidium japonicum Miq.		*Neolitsea*	Neolitsea aciculata (Blume) Koidz.
	Pternopetalum	Pternopetalum tanakae (Franch. & Sav.) Hand.-Mazz.			Neolitsea sericea (Blume) Koidz.
Aquifoliaceae	*Ilex*	Ilex crenata Thunb.	*Liliaceae*	*Chamaelirium*	Chamaelirium japonicum (Willd.) N.Tanaka
Araceae	*Arisaema*	Arisaema ringens (Thunb.) Schott		*Ophiopogon*	Ophiopogon jaburan G.Lodd.
	Pinellia	Pinellia tripartita (Blume) Schott		*Polygonatum*	Polygonatum cryptanthum H.L?v. & Vaniot
Araliaceae	*Dendropanax*	Dendropanax trifidus (Thunb.) Makino ex H.Hara	*Moraceae*	*Ficus*	Ficus erecta Thunb.
Asteraceae	*Ainsliaea*	Ainsliaea apiculata Sch.Bip.			Ficus erecta Thunb. f. sieboldii (Miq.) Corner
	Rhynchospermum	Rhynchospermum verticillatum Reinw.	*Myrsinaceae*	*Ardisia*	Ardisia crispa (Thunb.) A.DC.
	Wollastonia	Wollastonia dentata	*Oleaceae*	*Ligustrum*	Ligustrum japonicum Thunb.
Betulaceae	*Ostrya*	Ostrya japonica Sarg.			Ligustrum quihoui Carri?re
Boraginaceae	*Argusia*	Heliotropium sibiricum (L.) J.I.M.Melo	*Orchidaceae*	*Bletilla*	Bletilla striata (Thunb.) Rchb.f.
Campanulaceae	*Peracarpa*	Peracarpa carnosa (Wall.) Hook.f. & Thomson		*Cyrtosia*	Cyrtosia septentrionalis (Rchb.f.) Garay
	Wahlenbergia	Wahlenbergia marginata (Thunb.) A.DC.	*Pittosporaceae*	*Pittosporum*	Pittosporum tobira (Thunb.) W.T.Aiton
Caprifoliaceae	*Viburnum*	Viburnum odoratissimum Ker Gawl.	*Pteridaceae*	*Coniogramme*	Coniogramme japonica (Thunb.) Diels
Celastraceae	*Euonymus*	Euonymus nitidus Benth.		*Pteris*	Pteris cretica L.
Chloranthaceae	*Chloranthus*	Chloranthus fortunei (A.Gray) Solms			Pteris multifida Poir.

Commelinaceae	*Pollia*	Pollia japonica Thunb.	*Ranunculaceae*	*Actaea*	Actaea biternata (Siebold & Zucc.) Prantl
Dennstaedtiaceae	*Microlepia*	Microlepia strigosa (Thunb.) C.Presl			Actaea japonica Thunb.
Dryopteridaceae	*Dryopteris*	Dryopteris championii (Benth.) Christ ex Ching		*Adonis*	Adonis multiflora Nishikawa & Koji Ito
		Dryopteris fuscipes C.Chr.	*Rhamnaceae*	*Berchemia*	Berchemia floribunda (Wall.) Brongn.
	Polystichum	Polystichum lepidocaulon (Hook.) J.Sm.		*Sageretia*	Sageretia thea (Osbeck) M.C.Johnst.
Elaeagnaceae	*Elaeagnus*	Elaeagnus × submacrophylla Servett.	*Rosaceae*	*Rhaphiolepis*	Rhaphiolepis indica (L.) Lindl. ex Ker var. umbellata (Thunb. ex Murray)
		Elaeagnus glabra Thunb.		*Rubus*	Rubus corchorifolius L.f.
Ericaceae	*Vaccinium*	Vaccinium bracteatum Thunb.			Rubus hirsutus Thunb.
Euphorbiaceae	*Mallotus*	Mallotus japonicus (L.f.) M?ll.Arg.			Rubus ribisoideus Matsum.
	Mercurialis	Mercurialis leiocarpa Siebold & Zucc.	*Rubiaceae*	*Damnacanthus*	Damnacanthus indicus C.F.Gaertn.
Fabaceae	*Caesalpinia*	Caesalpinia decapetala (Roth) Alston	*Rutaceae*	*Zanthoxylum*	Zanthoxylum ailanthoides Siebold & Zucc.
	Dunbaria	Dunbaria villosa (Thunb.) Makino	*Sabiaceae*	*Meliosma*	Meliosma myriantha Siebold & Zucc.
	Desmodium	Ohwia caudata (Thunb.) H.Ohashi	*Schisandraceae*	*Kadsura*	Kadsura japonica (L.) Dunal
	Rhynchosia	Rhynchosia acuminatifolia Makino		*Schisandra*	Schisandra repanda (Siebold & Zucc.) Radlk.
		Rhynchosia volubilis Lour.	*Solanaceae*	*Scopolia*	Scopolia parviflora (Dunn) Nakai
	Wisteriopsis	Wisteriopsis japonica (Siebold & Zucc.) J.Compton & Schrire	*Theaceae*	*Cleyera*	Cleyera japonica Thunb.
	Castanopsis	Castanopsis sieboldii (Makino) Hatus. ex T.Yamaz. & Mashiba		*Eurya*	Eurya emarginata (Thunb.) Makino
	Quercus	Quercus acuta Thunb.	*Thymelaeaceae*	*Daphne*	Daphne kiusiana Miq.
		Quercus glauca Thunb.		*Wikstroemia*	Wikstroemia genkwa (Siebold & Zucc.) Domke
		Quercus myrsinifolia Blume			Wikstroemia trichotoma (Thunb.) Makino
Flacourtiaceae	*Idesia*	Idesia polycarpa Maxim.	*Urticaceae*	*Boehmeria*	Boehmeria holosericea Blume
Fumariaceae	*Corydalis*	Corydalis decumbens (Thunb.) Pers.		*Nanocnide*	Nanocnide japonica Blume
		Corydalis incisa (Thunb.) Pers.	*Verbenaceae*	*Callicarpa*	Callicarpa mollis Siebold & Zucc.
Gleicheniaceae	*Dicranopteris*	Dicranopteris pedata (Houtt.) Nakaike		*Verbena*	Verbena officinalis L.
Hamamelidaceae	*Distylium*	Distylium racemosum Siebold & Zucc.	*Viscaceae*	*Korthalsella*	Korthalsella japonica (Thunb.) Engl.
Juncaceae	*Juncus*	Juncus setchuensis Buchenau			

Supplementary Table 2. Environmental variables for SDMs

Num.	Class	Variable	Original resolution	Source
1	Bioclimate	Annual mean temperature (BIO1)	30 arc sec	Chelsa database (https://chelsa-climate.org/)
2		Mean diurnal range (BIO2)		
3		Isothermality (BIO3)		
4		Temperature seasonality (BIO4)		
5		Max temperature of warmest month (BIO5)		
6		Min temperature of coldest month (BIO6)		
7		Temperature annual range (BIO7)		
8		Mean temperature of wettest quarter (BIO8)		
9		Mean temperature of coldest quarter (BIO9)		
10		Mean temperature of warmest quarter (BIO10)		
11		Mean temperature of coldest quarter (BIO11)		
12		Annual precipitation (BIO12)		
13		Precipitation of wettest month (BIO13)		
14		Precipitation of driest month (BIO14)		
15		Precipitation seasonality (BIO15)		
16		Precipitation of wettest quarter (BIO16)		
17		Precipitation of driest quarter (BIO17)		
18		Precipitation of warmest quarter (BIO18)		
19		Precipitation of coldest quarter (BIO19)		
20		Köppen-Geiger climate classification		

21		Modified Köppen-Geiger climate classification 1		
22		Modified Köppen-Geiger climate classification 2		
23		Modified Köppen-Geiger climate classification 3		
24		Modified Köppen-Geiger climate classification 4		
25		Accumulated precipiation amount on growing season days TREELIM		
26		Mean temperature of the growing season TREELIM		
27		Net primary productivity		
28	Soil	Bulk density	250m	Soilgrids database (https://soilgrids.org/)
29		Volumetric fraction of quartz		
30		Organic carbon density		
31		Soil pH		
32		Proportion of sand particles		
33		Proportion of silt particles		
34		Soil organic carbon		
35	Landform	Altitude	90m	NASA SRTM90 (https://cmr.earthdata.nasa.gov/)
36		Roughness		
37		Solar radiation		
38		Topographic wetness index (TWI)		

Supplementary Table 3. Ecological interpretation of retained PCA axes. This table presents the variance explained, cumulative variance, and top contributing environmental variables for each retained PCA axis, together with an ecologically interpretable description and axis alias. The retained axes capture major climatic, edaphic, and topographic gradients while reducing multicollinearity among original predictors.

PC	Variance explained(%)	Cumulative(%)	Top 5 contributing variables	Variable code	Contribution rate(%)	Ecological interpretation	Axis alias
PC1	42.93	42.93	Soil organic carbon density	OCD	6.22	Soil fertility and chemical properties interacting with baseline thermal conditions	Soil fertility-thermal gradient
			Bulk density	BDOD	6.1		
			Total nitrogen	Nitrogen	5.68		
			Soil pH	PHH2O	5.59		
			Annual mean temperature	BIO1	5.47		
PC2	17.69	60.62	Annual temperature range	BIO7	18.11	Thermal seasonality and continentality distinguishing oceanic and inland regimes	Thermal seasonality gradient
			Temperature seasonality	BIO4	17.63		
			Minimum temperature of coldest month	BIO6	6.45		
			Mean temperature of driest quarter	BIO9	5.6		
			Mean temperature of coldest quarter	BIO11	5.48		
PC3	13.62	74.24	Elevation	Elevation	17.89	Elevation-linked thermal stability and diurnal temperature structure	Elevational thermal stability gradient
			Isothermality	BIO3	10.08		
			Mean diurnal temperature range	BIO2	7.47		
			Mean temperature of wettest quarter	BIO8	7.11		
			Mean temperature of warmest quarter	BIO10	6.85		
PC4	6.86	81.11	Precipitation seasonality	BIO15	25.55	Seasonal precipitation regime modulated by soil texture and moisture retention	Moisture seasonality-soil texture gradient
			Silt content	Silt	13.76		
			Sand content	Sand	10.39		
			Precipitation of coldest quarter	BIO19	9.97		
			Precipitation of driest month	BIO14	8.31		
PC5	5.47	86.58	Sand content	Sand	33.42	Soil texture and precipitation extremes influencing soil water dynamics	Soil texture-moisture gradient
			Silt content	Silt	32.28		
			Precipitation seasonality	BIO15	63.48		
			Precipitation of driest month	BIO14	3.95		
			Precipitation of driest quarter	BIO17	3.87		
PC6	3.58	90.17	Slope	Slop	22.15	Topographic complexity and surface heterogeneity shaping microclimatic variability	Topographic complexity gradient
			Terrain roughness	Rough	20.97		
			Coarse fragment volume	CFVO	16.87		
			Mean temperature of wettest quarter	BIO8	3.14		
			Mean temperature of warmest quarter	BIO10	3.04		

Supplementary Table 4. Model performance was evaluated using Kappa, ROC, and TSS through 5-fold cross-validation. The table summarizes average sensitivity, specificity, and cutoff thresholds for the ensemble model (top) and its component algorithms (bottom) across all species.

Algorithm	Metric	Average sensitivity (%)	Standard deviation of sensitivity	Average specificity (%)	Standard deviation of specificity	Average cutoff	Standard deviation of cutoff
EMwmean	KAPPA	83.4	7.1	98.8	1.5	627.1	83.2
EMwmean	ROC	93.5	4.6	94.6	3.6	407.4	141.6
EMwmean	TSS	93.5	4.5	94.4	3.8	405.6	141.2

Algorithm	Metric	Average sensitivity (%)	Standard deviation of sensitivity	Average specificity (%)	Standard deviation of specificity	Average cutoff	Standard deviation of cutoff
ANN	KAPPA	83.9	16.3	95.3	5.9	722.7	191.2
ANN	ROC	95.6	3.8	90.6	10.2	566.8	180.6
ANN	TSS	95.6	3.8	90.6	10.2	566.3	181
CTA	KAPPA	93.9	5.9	91.5	8.6	596.6	183.5
CTA	ROC	96.2	4.2	90.7	8.8	465.2	55.6
CTA	TSS	96.2	4.2	90.7	8.8	462.4	55.3
GAM	KAPPA	99.6	1.6	99.7	1.7	508.3	49.6
GAM	ROC	99.8	1.2	99.6	1.9	507.9	37.2
GAM	TSS	99.8	1.2	99.6	1.9	504.4	38.5
GBM	KAPPA	96.6	5.5	98.4	2.0	586.6	97.5
GBM	ROC	99.7	1.0	97.5	3.7	514.3	60.8
GBM	TSS	99.7	1.0	97.5	3.8	513.1	60.2
MARS	KAPPA	92.9	10.2	97.4	3.1	710.9	162.7
MARS	ROC	98.7	2.8	95.1	6.7	587.2	161
MARS	TSS	98.7	2.8	95.0	6.8	581.0	157.6
RF	KAPPA	99.1	1.7	99.8	0.2	407.7	66.6
RF	ROC	100.0	0.1	99.6	0.3	380.0	56.0
RF	TSS	100.0	0.1	99.6	0.3	378.1	55.6

Supplementary Table 5. Summary of environmental characteristics by cluster. Mean, standard deviation, and z-scores for latitude, longitude, elevation, and sea distance are provided for each of the nine species clusters. Brief labels describe each cluster's dominant geographic and topographic traits.

**Cluster**	**Latitude (°)**		**Longitude (°)**		**Elevation (m)**		**Sea Distance (km)**		**n**	**Main Characteristics**	**Ecological Name**
	mean (sd)	z	mean (sd)	z	mean (sd)	z	mean (sd)	z			
1	35.06 (0.28)	–0.98	128.35 (0.48)	0.71	90.3 (78.1)	–0.59	8.7 (11.2)	–0.81	123	Lowland, southeastern coastal zone	Southern Coastal Lowland
2	34.59 (0.60)	–1.38	126.64 (0.40)	–1.11	86.5 (91.3)	–0.61	5.1 (6.6)	–0.92	190	Southernmost, strongly coastal	Southern Maritime Zone
3	36.57 (0.84)	0.29	129.08 (0.28)	1.48	204.7 (137.9)	–0.10	18.8 (16.8)	–0.49	131	Mid-latitude, eastern inland	Eastern Inland Lowland
4	37.02 (0.57)	0.67	126.58 (0.47)	–1.17	57.5 (49.5)	–0.73	9.2 (9.5)	–0.79	156	Western lowland near coast	Western Coastal Plain
5	35.60 (0.52)	–0.53	127.72 (0.53)	0.04	606.2 (166.7)	1.61	57.4 (21.4)	0.72	64	Central highland zone	Central Highland Zone
6	35.86 (0.57)	–0.31	127.27 (0.34)	–0.44	158.9 (110.7)	–0.30	42.0 (12.8)	0.23	114	Transitional inland zone	Central Inland Transition
7	36.51 (0.45)	0.24	128.18 (0.34)	0.53	251.6 (142.8)	0.1	85.7 (15.8)	1.6	166	Eastern inland, far from coast	Eastern Inland Upland
8	37.53 (0.50)	1.11	128.55 (0.43)	0.92	739.6 (163.0)	2.18	38.6 (20.0)	0.13	102	Highest elevation, northern mountains	Northern Montane Zone
9	37.71 (0.39)	1.25	127.53 (0.34)	–0.17	244.1 (142.1)	0.07	63.3 (15.9)	0.9	124	Northern upland, inland region	Northern Inland Upland

Supplementary Table 6. Metrics of richness-weighted 50% kernel density hotspot regions across time periods. Mean and standard deviation (SD) of total area (km^2), median area (km^2), and number of 50% kernel density estimation (KDE50) polygons are summarized for the current period (1980–2010) and three future periods (2010–2040, 2040–2070, 2070–2100). Values for future periods represent averages across three climate scenarios (SSP1–2.6, SSP3–7.0, SSP5–8.5). SDs are not provided for the current period.

Period	Mean Total Area (km^2)	SD Total Area (km^2)	Mean Median Area (km^2)	SD Median Area (km^2)	Mean n
**Current**	47,184.7	–	287.4	–	10.0
**2010–2040**	47,497.7	642.5	1,053.8	400.3	8.7
**2040–2070**	48,195.2	395.5	1,280.0	530.3	7.3
**2070–2100**	49,652.4	1,100.2	1,438.4	514.4	7.0

Supplementary Table 7. Summary of spatial and environmental attributes of KDE50 hotspot regions by scenario and time period. This table presents the estimated area, centroid location, northernmost extent, mean elevation, and mean distance to the coastline for the 50% kernel density (KDE50) hotspot zones of southern species across three SSP scenarios (126, 370, 585) and four time periods (Current, 1140, 4170, 7100). It also reports the 10th, median, and 90th percentile latitudes to show the internal latitudinal distribution of hotspot regions. These metrics support the analysis of spatial redistribution under climate change.

Scenario	Period	KDE50 Area (km^2)	Centroid Lon.	Centroid Lat.	Northern Edge Lat.	Mean Elevation (m)	Mean Sea Distance (m)	Lat. 10th %tile	Lat. Med.	Lat. 90th %tile
126	Current	47,184.70	127.5844	35.5776	38.2944	222.84	27,281.09	34.4602	35.3821	37.1348
126	1140	48,091.40	127.7163	35.7995	38.4354	278.25	30,099.12	34.4849	35.4278	37.6896
126	4170	47,979.72	127.7546	35.6685	38.4818	274.83	26,761.94	34.4683	35.4014	37.3701
126	7100	48,383.19	127.7133	35.6289	38.4466	258.74	27,993.99	34.4791	35.4130	36.9873
370	Current	47,184.70	127.5844	35.5776	38.2944	222.84	27,281.09	34.4602	35.3821	37.1348
370	1140	47,586.19	127.7691	35.6821	38.4740	273.65	27,018.42	34.4603	35.3911	37.4994
370	4170	47,954.21	127.7591	35.6770	38.3746	264.22	26,465.46	34.4842	35.4086	37.4858
370	7100	50,062.36	127.7654	35.9152	38.4757	310.51	31,286.10	34.5208	35.4725	37.8298
585	Current	47,184.70	127.5844	35.5776	38.2944	222.84	27,281.09	34.4602	35.3821	37.1348
585	1140	46,815.48	127.7370	35.6124	38.3704	263.31	27,381.31	34.4676	35.3717	37.2517
585	4170	48,651.59	127.7814	35.6864	38.4575	277.23	27,430.17	34.4888	35.4227	37.3469
585	7100	50,240.27	127.7198	35.7999	38.4100	289.79	30,127.00	34.5202	35.4939	37.4855

Supplementary Table 8. Effects of cluster and period on species composition (PERMANOVA) with dispersion diagnostics (PERMDISP). Global PERMANOVA assessed the effects of cluster and period on Bray–Curtis dissimilarities (4,999 permutations). By-cluster PERMANOVA tested the period effect within each cluster (1,999 permutations). PERMDISP evaluated the heterogeneity of multivariate dispersion among periods (999 permutations). Values are reported as medians across 30 resamples; *p*-values are formatted as "<0.001", and significance is denoted as ** p<0.01, *** p<0.001. R^2 is not applicable to PERMDISP.

Section	Term	R^2 (median, IQR)	F (median, IQR)	*p*-value	Sig.
**Global PERMANOVA**	cluster	0.364 (0.009)	57.79 (3.00)	<0.001	***
	period	0.083 (0.008)	35.70 (3.68)	<0.001	***
**By-cluster PERMANOVA (period)**	C1(Cluster1)	0.084 (0.015)	2.32 (0.46)	<0.001	***
	C2	0.069 (0.021)	1.88 (0.61)	<0.001	***
	C3	0.137 (0.030)	4.01 (1.01)	<0.001	***
	C4	0.114 (0.032)	3.26 (1.01)	<0.001	***
	C5	0.265 (0.041)	9.14 (1.89)	<0.001	***
	C6	0.190 (0.028)	5.93 (1.11)	<0.001	***
	C7	0.205 (0.036)	6.54 (1.46)	<0.001	***
	C8	0.333 (0.059)	12.66 (3.39)	<0.001	***
	C9	0.386 (0.064)	15.93 (4.41)	<0.001	***
**PERMDISP (periods)**	ANOVA	—	41.16 (10.11)	<0.001	***
	Perm test	—	41.16 (10.11)	0.001	**

Supplementary Table 9. Cluster-wise alignment between compositional trajectories and environmental gradients. Results from PCoA–envfit analysis based on Bray–Curtis dissimilarities quantify how cluster-wise centroid trajectories align with environmental vectors. The Driver variable denotes the best-aligned predictor for each cluster. Alignment types are categorized as aligned (→), oblique (↗), or opposed (←) based on the sign of cos Δθ, with the projected shift (L·cos Δθ) indicating directional displacement along the driver. Positive values of cos Δθ imply gradient-following shifts (e.g., northward movement with latitude), while negative values indicate deviation or movement in the opposite direction.

Cluster	Driver Variable	Alignment Type	cos Δθ (IQR)	Projected Shift (L·cos Δθ)	Driver r^2	Driver *p*
1	y	→ aligned	0.86 (0.19)	0.047	0.521	<0.001
2	y	→ aligned	0.97 (0.10)	0.032	0.521	<0.001
3	Isothermality	↗ oblique	–0.45 (0.21)	–0.100	0.186	<0.001
4	Isothermality	↗ oblique	–0.36 (0.54)	–0.067	0.186	<0.001
5	y	↗ oblique	0.28 (0.19)	0.048	0.521	<0.001
6	y	↗ oblique	0.27 (0.27)	0.048	0.521	<0.001
7	Isothermality	← opposed	–0.54 (0.22)	–0.192	0.186	<0.001
8	Isothermality	← opposed	–0.53 (0.22)	–0.182	0.186	<0.001
9	Isothermality	← opposed	–0.65 (0.15)	–0.276	0.186	<0.001

Supplementary Table 10. Changes in Whittaker climate-space ellipse area and overlap. To evaluate shifts in climate space between the current and 7100 periods, 95% covariance ellipses were fitted in the space defined by Mean Annual Temperature (MAT) and Mean Annual Precipitation (MAP). Area change is reported as ΔArea (%), and spatial overlap is measured using Overlap (Jaccard) and Overlap (Min-based). Due to mixed units (°C·cm), interpretation emphasizes relative change and overlap metrics.

Level	Area (Current, °C·cm)	Area (Future, °C·cm)	ΔArea (%)	Overlap (Jaccard)	Overlap (Min-based)
95%	702.05	733.72	4.5	0.183	0.317

Supplementary Table 11. Linkages between analytical components and the Kunming–Montreal Global Biodiversity Framework (GBF)

Analytical component	Description	Relevant GBF target(s)	Conservation relevance
Species distribution modeling (SDMs)	Projected species-level distribution changes under multiple climate scenarios	Target 2, Target 3	Identifies areas of future habitat contraction/expansion relevant for restoration and protection planning
Static environmental clustering	Delineation of ecologically coherent, scenario-invariant spatial zones	Target 3	Supports spatially coherent conservation zoning beyond static administrative boundaries
Richness-weighted KDE (core response zones)	Identification of spatial hotspots of persistent species richness	Target 2, Target 3	Highlights potential climate refugia and priority areas for long-term protection
Ordination-based trajectory analysis	Quantification of magnitude, direction, and variability of compositional change	Target 2	Reveals where and how community reorganization is occurring under climate change
Environmental alignment (directionality metrics)	Assessment of whether compositional shifts follow climatic gradients	Target 2	Distinguishes climate-tracking dynamics from destabilized or novel ecosystem trajectories
Temporal turnover decomposition	Identification of periods with strongest compositional change	Target 2	Informs timing of intervention and adaptive management priorities

* Note: GBF Target 2 aims to restore at least 30% of degraded terrestrial, inland water, coastal, and marine ecosystems by 2030, enhancing ecosystem integrity and resilience. GBF Target 3 focuses on conserving at least 30% of land and sea areas through effectively managed, ecologically representative, and well-connected systems of protected areas and other effective area-based conservation measures.
